# Integrated multi-omics reveals stable microbe–metabolite relationships in pediatric celiac disease

**DOI:** 10.3389/fmicb.2026.1866060

**Published:** 2026-07-27

**Authors:** Peter J. Prendergast, Alan Aitchison, Shaun S. C. Ho, Vanessa K. Morris, Christoph Göbl, Renwick C. J. Dobson, Andrew S. Day, Olivia J. Ogilvie

**Affiliations:** 1 School of Biological Sciences, University of Canterbury, Christchurch, New Zealand; 2 Biomolecular Interaction Centre, University of Canterbury, Christchurch, New Zealand; 3 Riddet Institute, University of Canterbury, Christchurch, New Zealand; 4 Department of Paediatrics and Child Health, The University of Otago Christchurch, New Zealand; 5 Department of Gastroenterology and Clinical Nutrition, The Royal Children’s Hospital, Melbourne, Parkville, VIC, Australia; 6 Murdoch Children’s Research Institute, Parkville, VIC, Australia; 7 Department of Paediatrics, University of Melbourne, Parkville, VIC, Australia; 8 Mātai Hāora - Centre for Redox Biology and Medicine, The University of Otago Christchurch, New Zealand; 9 Department of Biochemistry and Pharmacology, Bio21 Molecular Science and Biotechnology Institute, University of Melbourne, Parkville, VIC, Australia

**Keywords:** *Bacteroides*, *Bilophila*, celiac disease, gluten-free diet, gut microbiome, metabolomics, multiomics, short-chain fatty acids

## Abstract

Celiac disease is a lifelong immune-mediated disease triggered by dietary gluten and managed exclusively through a strict gluten-free diet. Differences in the gut microbiome are consistently observed in celiac disease, yet the associated metabolic consequences remain insufficiently defined. This study evaluated the stool microbiome-metabolome relationship in children from Canterbury, New Zealand, a region with a notably high prevalence of celiac disease (∼ 1 in 82). Eleven children with untreated celiac disease and 10 controls were assessed, with nine children resampled after 6 months of gluten-free diet adherence. Stool samples were subjected to 16S rRNA sequencing, targeted and untargeted metabolomic profiling, and multi-omics integration using cross-block and machine-learning approaches. Celiac disease was characterized by consistent reproducible changes in a small set of resampled-stable taxa and metabolites, rather than a uniform shift. The most prominent finding was rearrangement within *Bacteroides* rather than a uniform shift: *B. ovatus* increased reproducibly in both untreated and treated disease, while integration analyses associated *B. fragilis* with celiac disease and *B. vulgatus* with controls. Metabolite changes were observed in two linked axes: a butyrate-led fermentation signature and a redox and organic acid signature, both elevated in untreated disease and shifting toward control levels after 6 months of gluten-free diet. Recovery was partial, and the diet imposed its own signature, notably an increase in the fat-responsive pathobiont *Bilophila wadsworthia*. Machine learning discrimination was modest (ROC-AUC = 0.66–0.68, PR-AUC = 0.73–0.79), but the microbiome and metabolome aligned along one reproducible cross-omic axis. The results support prioritizing a concise list of reproducible candidate features for mechanistic validation and longitudinal treatment monitoring in more extensive cohorts, rather than for immediate diagnostic application.

## Introduction

Celiac disease (CeD) is an immune-mediated disorder that affects 1–2% of the global population, with increasing prevalence and substantial rates of underdiagnosis (Catassi et al., 2022; King et al., 2020; Kvamme et al., 2022; Rubio-Tapia et al., 2012; Singh et al., 2018). CeD is defined by an immune response to dietary gluten, resulting in small-intestinal inflammation, villous atrophy, and a broad range of intestinal and extra-intestinal symptoms (Catassi et al., 2022; Lindfors et al., 2019). The sole established treatment for CeD is strict adherence to a gluten-free diet (GFD). While the GFD can effectively control the disease in some individuals, it is an imperfect therapy; symptoms persist in a significant proportion of patients (22–25%), and maintaining long-term dietary adherence imposes nutritional and psychosocial burdens (Gessaroli et al., 2023; Vici et al., 2016). Complete mucosal healing often requires a longer duration than symptomatic or serological improvement, frequently taking 1–2 years or more (Catassi et al., 2022; Lindfors et al., 2019). In certain cases, mucosal healing remains incomplete despite ongoing treatment (Catassi et al., 2022; Lindfors et al., 2019). These limitations of the current therapeutic approach have intensified interest in identifying upstream disease triggers and modifiable mechanisms beyond downstream inflammation (Büchold et al., 2022; Pultz et al., 2021).

The pathogenesis of CeD is strongly associated with the HLA-DQ2 and HLA-DQ8 gene variants, which are present in 30–40% of the global population (Catassi et al., 2022; King et al., 2020; Megiorni and Pizzuti, 2012; Sciurti et al., 2018; Singh et al., 2018). However, genetic susceptibility alone does not account for the fact that the disease develops in only a minority of genetically at-risk individuals, suggesting that environmental and host-microbial interactions contribute to disease initiation and clinical heterogeneity (Megiorni and Pizzuti, 2012; Sciurti et al., 2018). The gut microbiome is a plausible contributor to the loss of tolerance to gluten, as it interacts directly with dietary antigens, the epithelial barrier, and mucosal immunity (Galipeau and Verdu, 2022). Furthermore, microbial metabolism produces bioactive small molecules that influence the host immune response (Galipeau and Verdu, 2022).

Consistent with this, CeD onset is associated with shifts in microbial community structure, including changes in taxa linked to short-chain fatty acid (SCFA) production and mechanistic interactions between bacteria and gluten processing (Caminero et al., 2019; De Palma et al., 2009; Galipeau et al., 2015; Leonard et al., 2021; Nadal et al., 2007; Rahmani et al., 2024). However, taxonomic microbial signatures in individuals with CeD vary across cohorts and populations, and community composition alone provides limited insight into the mechanisms of host-microbe interactions (Galipeau and Verdu, 2022; Leonard et al., 2020; Leonard et al., 2021; Prendergast et al., 2026). Prospective multi-omics studies in CeD, such as birth cohorts collecting longitudinal stool and blood samples, have combined microbiome profiling with metabolomics to identify microbial features and metabolites detectable before seroconversion or diagnosis (Girdhar et al., 2023; Leonard et al., 2020; Leonard et al., 2021; Prendergast et al., 2026). Nevertheless, cross-cohort generalizability remains limited (Prendergast et al., 2026). Functional readouts that capture microbial and host metabolic activity are, therefore, likely necessary for identifying reproducible, biologically interpretable pathways.

Canterbury provides a compelling setting for population-specific mechanistic profiling of CeD, with a prevalence of at least 1 in 82 individuals (Cook et al., 2000), representing a substantially higher disease burden than global estimates or other local studies (Johnston et al., 1998; Laass et al., 2015; Singh et al., 2018). The region’s disease profile was first established through a 30-year audit (1970–1999) that identified 416 individuals with biopsy-confirmed CeD (Cook et al., 2004). This audit documented a marked increase in recognized incidence from 1.4 per 100,000 (1970–1972) to 12.9 per 100,000 (1997–1999), with increases first observed in adults (mid 1980s) and later in children (early 1990s) (Cook et al., 2004). The authors attributed this rise in incidence to changes in clinical practice, including the adoption of endoscopic duodenal biopsy (adults from 1989; children from 1992 to 1994) and the introduction of more specific serological tests (anti-gliadin antibodies in 1987; endomysial antibodies in 1994) (Cook et al., 2004). More recent pediatric data from Canterbury reinforce this trend, showing both increased detection and greater clinical diversity: between 2000 and 2010, 263 children were diagnosed, with annual diagnoses rising from 13 to 31. Of these, 21.6% were identified through screening of high-risk groups, such as first-degree family members, many of whom were asymptomatic at diagnosis (Kho et al., 2015).

Collectively, these Canterbury studies demonstrate a high local disease burden, rising incidence over time, and a substantial proportion of non-classical or screen-detected cases. However, these investigations predate modern integrated analyses that connect microbial ecology to functional biochemical outputs. Moreover, few multi-omics studies have examined the interaction between the gut microbiome and CeD. Additional research is therefore required to determine whether multi-omics approaches can reveal functional interactions between the gut microbiome and its metabolic outputs.

To address this gap, a Canterbury-specific multi-omics framework was implemented (Supplementary Figure 1). This approach employed 16S ribosomal RNA (rRNA) sequencing to define gut microbiome composition and combined both discovery- and targeted-mode metabolomics to identify patterns of metabolic change. The study design enabled analysis during untreated CeD and early post-GFD recovery. Investigating multi-omics dynamics in a high-disease-burden population, such as Canterbury, is critical, as it may uncover disease attributes not observed in less affected populations and provide actionable insights into disease pathology (Alemu et al., 2025).

## Materials and methods

### Sample collection and inclusion criteria

Children presenting to Christchurch Hospital with suspected CeD were recruited during assessment. At the baseline visit, participants provided stool and blood samples for downstream analysis, which were frozen and stored at -80 °C. CeD serology: anti-tissue transglutaminase IgA (anti-tTG IgA) and anti-endomysial IgA antibodies were assessed as part of the evaluation; a duodenal biopsy was obtained from all participants.

Participants were assigned to one of three groups based on pre-established criteria. Untreated CeD cases were required to have CeD confirmed by duodenal biopsy at the baseline (pre-treatment) visit. Treated CeD participants were the same children resampled after 6 months on a GFD and had to demonstrate clinical adherence, shown by a decrease in anti-tTG IgA compared with baseline. Control participants were children who visited the same clinic for diagnostic endoscopic investigation and in whom CeD was ruled out, requiring negative CeD serology (negative anti-tTG IgA and anti-endomysial IgA antibodies) along with a histologically normal duodenal biopsy. Controls were therefore non-CeD clinical comparators rather than healthy population controls, as they were examined for other clinical indications.

To minimize the influence of unrelated conditions on the gut microbiome, all participants, including controls, were required to have no known gastrointestinal or intestinal diseases such as Crohn’s disease or ulcerative colitis. Controls also needed to show no histological signs of enteropathy on duodenal biopsy. Six children initially considered as potential controls were excluded after testing positive for CeD serology, ensuring the control group was both serologically and histologically clean.

### 16 S rRNA sequencing

Bacterial DNA was extracted from stool samples using the QIAamp Fast DNA Stool Mini Kit and quantified by Nanodrop. Bacterial DNA was profiled by 16S rRNA gene amplicon sequencing of the V3-V4 region via the Massey Genome Service (Massey University, New Zealand), based on the dual-index sequencing approach (Kozich et al., 2013; Caporaso et al., 2011). Amplicons were generated using the following primers: 341F V3: (CCTACGGGAGGCAGCAG) and 806R V4: (GGACTACHVGGGTWTCTAAT) (Caporaso et al., 2011; López-Gutiérrez et al., 2004). Dual-index amplicon libraries were sequenced on an Illumina MiSeq run using 2 x 250 bp paired-end chemistry.

### 16 S rRNA data processing

Raw paired-end 16S rRNA reads were initially quality checked using FastQC (v0.11.9) (Simon Andrews et al., 2010), and adapters were removed using Trimmomatic (v0.39) (Bolger et al., 2014). Amplicon sequence variants were deionised by DADA2 (v1.36.0) (Callahan et al., 2016a), and taxonomic classification was assigned using the SILVA reference database (v138.1) (Quast et al., 2013) following an established pipeline (Callahan et al., 2016b). R (v4.4.0) and phyloseq (v1.48.0) (McMurdie and Holmes, 2013) were used for downstream processing and analysis. Full code, including filtering criteria and generation of analysis-ready datasets, is provided in the accompanying GitHub script.

### Alpha diversity and phylogenetic diversity

Alpha diversity was calculated in R from the aforementioned phyloseq objects using the microbiome package (v1.26.0) (Shetty and Lahti, 2019), and seven metrics were analyzed (observed richness, Shannon, Fisher, Bulla, Simpson, dominance, and rare-abundance indices). Each metric was analyzed on a log_2_ scale. Linear models were fitted for each metric, with disease status as the primary factor. Unpaired comparisons were fitted using:


$$l\,o\,g_{2}\,\left(m\,e\,t\,r\,i\,c\right)∼d\,i\,s\,e\,a\,s\,e+s\,t\,a\,t\,u\,s+r\,e\,a\,d\,d\,e\,p\,t\,h+a\,g\,e+s\,e\,x$$

For paired comparisons (treated vs. untreated CeD), linear mixed-effects models (LMMs) were fitted with a random intercept for subject and adjustment for read depth:


$$l\,o\,g_{2}\,\left(m\,e\,t\,r\,i\,c\right)∼s\,t\,a\,t\,u\,s+r\,e\,a\,d\,d\,e\,p\,t\,h\,\left(1|S\,u\,b\,j\,e\,c\,t\right)$$

Faith’s phylogenetic diversity (PD) was calculated using the Picante (v1.8.2) (Kembel et al., 2010), lme4 (v1.1.35.5) (Bates et al., 2015), lmerTest (v3.1.3) (Kuznetsova et al., 2017), and dplyr packages (v1.1.4) (Wickham et al., 2023). Faith’s PD was modeled on its raw (untransformed) scale: unpaired comparisons used a linear model with disease status as the main factor and age and sex as covariates, while the paired comparison employed a LMM with diet status, an age covariate, and a random intercept for each subject. Read depth was not included as a covariate in the PD models.

Alpha diversity and Faith’s PD models were re-fitted across 500 bootstrap resamples, using subject-level resampling for paired data. Diversity effects were considered stable when the bootstrap estimates retained the observed direction and the 95% bootstrap interval remained on one side of zero. Per-metric *p*-values are reported unadjusted, as all raw *p*-values exceeded 0.05, multiple-testing correction would not alter the conclusion of no significant alpha or phylogenetic diversity differences.

### Beta diversity

Microbiome beta diversity was calculated and analyzed using the following packages: vegan (v2.6.6.1) (Oksanen et al., 2013), compositions (v2.0.8) (Van den Boogaart and Tolosana-Delgado, 2008), and pairwiseAdonis (v0.4.1) (Arbizu, 2020) using the Aitchison distance on compositional count data. Counts were transformed using a centered log-ratio (CLR) approach, with a pseudo-count of 1e-6 to accommodate features with zeros. Group-level variation was tested using PERMANOVA (4,999 permutations) with disease status as the primary factor, and host covariates (age, sex) included in the model. For the paired comparison, sex was omitted because it is invariant, giving a model of group plus age, and significance was assessed using exact subject-restricted permutations that swapped the two timepoints within each subject. Multivariate homogeneity of group dispersions was additionally evaluated using a permutation-based dispersion test.

To examine whether age or sex influenced the effect of the disease group on community composition, pre-specified sensitivity analyses added two interaction terms: Group × Sex and Group × Age. These were added to each PERMANOVA model sequentially. All interaction terms were placed last and tested at the margin. The same interaction structure was used for alpha diversity, Faith’s PD linear models, and metabolomics LMMs. Likelihood-ratio tests compared base models with models including interactions. The Benjamini-Hochberg (BH) correction was used to control the false discovery rate (FDR) and was applied to all interaction tests within each section.

### Phylum-level analysis

Phylum-level analysis was done using the following packages: tidyr (v1.3.1) (Wickham et al., 2019), and broom (v1.0.8) (Robinson, 2014). Phylum-level relative abundances were calculated from the prevalence-filtered phyloseq objects, aggregating amplicon sequence variants by phylum. The top five were analyzed (Bacteroidetes, Firmicutes, Proteobacteria, Actinobacteria, and Verrucomicrobia). Relative abundances for these phyla were renormalized to within-sample proportions across the selected phyla and arcsine square-root transformed prior to modeling. For unpaired comparisons, phylum abundances were tested using linear models:


$$\arcsin\left(\sqrt{A\,b\,u\,n\,d\,a\,n\,c\,e}\right)∼d\,i\,s\,e\,a\,s\,e+s\,t\,a\,t\,u\,s+a\,g\,e+s\,e\,x$$

Paired LMMs were fitted using:


$$\arcsin\left(\sqrt{A\,b\,u\,n\,d\,a\,n\,c\,e}\right)∼d\,i\,e\,t\,s\,t\,a\,t\,u\,s+a\,g\,e+\left(1|S\,u\,b\,j\,e\,c\,t\right)$$

Multiple testing was controlled using the BH FDR correction, with *q* < 0.05 considered significant within each model term. As a sensitivity analysis, each phylum contrast was re-estimated with 500 bootstrap resamples (subject-level resampling for the paired comparison). Phylum effects were regarded as stable only when the observed direction was maintained with high bootstrap sign consistency and the 95% bootstrap interval excluded zero.

### Differential abundance

Differential abundance was analyzed at both the genus and species level using ANCOM-BC2 (v2.6.0) (Lin and Peddada, 2024). For unpaired comparisons, ANCOM-BC2 was fit using the following model:


d⁢i⁢s⁢e⁢a⁢s⁢e⁢s⁢t⁢a⁢t⁢u⁢s⁢(p⁢r⁢i⁢m⁢a⁢r⁢y)+a⁢g⁢e+s⁢e⁢x⁢(f⁢i⁢x⁢e⁢d⁢e⁢f⁢f⁢e⁢c⁢t⁢s)

Paired comparisons were fitted using:


d⁢i⁢e⁢t⁢s⁢t⁢a⁢t⁢u⁢s⁢(p⁢r⁢i⁢m⁢a⁢r⁢y⁢f⁢i⁢x⁢e⁢d⁢e⁢f⁢f⁢e⁢c⁢t)+(1|S⁢u⁢b⁢j⁢e⁢c⁢t)⁢(r⁢a⁢n⁢d⁢o⁢m⁢i⁢n⁢t⁢e⁢r⁢c⁢e⁢p⁢t)

ANCOM-BC2 was run with structural zeroes adjusted and pseudo-count sensitivity enabled. Features were additionally filtered within ANCOM-BC2 using a prevalence cutoff of 15%. ANCOM-BC2 effect sizes are reported as bias-corrected log fold change (LFC). Probability values were adjusted within ANCOM-BC2 using BH FDR correction with significant features initially defined as *p* < 0.05; *q* < 0.05; and LFC ± 0.5.

To distinguish reproducible differential abundance signals from features that were only marginally significant, each ANCOM-BC2 contrast underwent a non-parametric bootstrap robustness analysis with 500 resamples, using a fixed seed for reproducibility. Resampling maintained the design of each contrast: unpaired contrasts were resampled with replacement within groups, whereas the paired (treated vs. untreated CeD) contrast was resampled at the subject level, making sure that both time points from a given child were kept together. The full ANCOM-BC2 model, prevalence/library filtering, and significance thresholds described earlier were reapplied to each resample. For each taxon, the bootstrap distribution was summarized by its selection frequency (the proportion of resamples where it met the observed significance criteria), as well as the median and 95% percentile interval of its LFC, and the median and 95% interval of its within-fit evidence rank (ranked by the ANCOM-BC2 test statistic *W* = LFC/*SE*). A taxon was considered bootstrap-robust only if it met all three criteria: a selection frequency of at least 0.55, a 95% bootstrap LFC interval excluding zero, and median evidence rank within the top 30% of testable features for that contrast (a relative threshold, as the number of testable features varies between contrasts). Robust taxa are emphasized as the primary findings, while the remaining BH FDR-significant but non-robust taxa are reported as a secondary, less-stable set.

### NMR collection, processing, and analysis

Stool samples were extracted in technical replicates (≥3 per individual; mostly 4, limited by sample availability) using 100 mg of sample per replicate, which were stored at -80 °C. The stool was suspended in phosphate-buffered saline (PBS; pH 7.35) at 1:5 (stool: buffer), combined with glass beads, and vortex-homogenized until fully homogenized. Samples were then centrifuged at 14,000 × g for 30 min at 4 °C, and the supernatant was collected. Sodium 3-trimethylsilyl propionate (TSP; 4 mM) was then added, and D_2_O at 10% v/v of the final fraction (600 mL total sample volume). One-dimensional ^1^H nuclear magnetic resonance (NMR) spectra were acquired on a Bruker 600 MHz spectrometer, operated by an Advance III console, equipped with a TXi triple-resonance probe with z-axis gradients. Samples were measured in 5 mm tubes (Bruker) and were stored at 4 °C between measurements using a SampleJet autosampler. For data acquisition, a standard 1-dimensional Carr-Purcell-Meiboom-Gill T2 filter experiment with an echo spacing of 32 ms between refocusing pulses was performed. A presaturation pulse of 2 s was applied for water suppression at 298 K, with 64 scans, a 4 s relaxation delay, a 16-ppm spectral width, and 32,768 complex data points acquired. Spectra were manually phase-corrected and referenced using TSP at 0.00 ppm. NMRProcFlow (v1.4) (Jacob et al., 2017) was used to apply intelligent binning and probabilistic quotient normalization. Normalized spectra bins were annotated using the Chenomx NMR Suite (v11.0) (Chenomx Inc, 2024) by matching peaks to the reference compound library. Bins were only identified as a single metabolite when a representative non-overlapping peak was identified (Supplementary Figure 2). Each identified metabolite/bin was then analyzed using LMMs:


M⁢e⁢t⁢a⁢b⁢o⁢l⁢i⁢t⁢e∼d⁢i⁢s⁢e⁢a⁢s⁢e⁢s⁢t⁢a⁢t⁢u⁢s+a⁢g⁢e+s⁢e⁢x+(1|S⁢a⁢m⁢p⁢l⁢e)

M⁢e⁢t⁢a⁢b⁢o⁢l⁢i⁢t⁢e∼d⁢i⁢e⁢t⁢s⁢t⁢a⁢t⁢u⁢s+a⁢g⁢e+s⁢e⁢x+(1|S⁢u⁢b⁢j⁢e⁢c⁢t/S⁢a⁢m⁢p⁢l⁢e)

Wald tests were employed to assess significance; *p* < 0.05 and *q* < 0.10 indicated significance under the BH FDR correction. As each stool sample contributed several technical replicates, NMR models were fitted at the replicate level with a random intercept for the biological sample in the unpaired comparisons and a nested subject/sample random intercept in the paired untreated-versus-treated comparison. To evaluate stability, these metabolite models were also re-estimated using 500 cluster bootstrap resamples, stratified by group for unpaired contrasts and resampled at the subject level for paired contrasts. Metabolites were considered bootstrap-stable only when the observed BH FDR-adjusted *q*-value remained ≤ 0.10 and the 95% bootstrap interval did not cross zero.

### GC-MS measurement and analysis

Stool samples were freeze-dried and analyzed by targeted gas chromatography-mass spectrometry (GC-MS) at the Mass Spectrometry Centre, The University of Auckland, New Zealand, using an Agilent Technologies GC7890 coupled to an MSD5975. Metabolite concentrations were normalized by sample weight before analysis. Each metabolite was then analyzed using an LMM with disease status as the fixed effect of interest, age and sex as covariates, and a subject-specific random intercept to account for repeated measures:


M⁢e⁢t⁢a⁢b⁢o⁢l⁢i⁢t⁢e∼d⁢i⁢s⁢e⁢a⁢s⁢e⁢s⁢t⁢a⁢t⁢u⁢s+a⁢g⁢e+s⁢e⁢x+(1|S⁢u⁢b⁢j⁢e⁢c⁢t)

Wald tests were used to compute *p*-values for disease status effects, and multiple testing across metabolites was controlled using BH FDR. Significantly different metabolites were defined by *p* < 0.05 and *q* < 0.10. The same 500-resample cluster bootstrap framework was then applied as a sensitivity analysis, with robust metabolites defined as those with observed *q* ≤ 0.10 and a 95% bootstrap interval entirely above or below zero.

### Community-level ordination

Overall community structure in the microbiome and metabolome was summarized with a shared ordination and permutation approach, applying it separately to each data type with vegan (v2.6.6.1) (Oksanen et al., 2013), compositions (v2.0.8) (Van den Boogaart and Tolosana-Delgado, 2008), and pairwiseAdonis (v0.4.1) (Arbizu, 2020). For each dataset, we created a sample-by-sample distance matrix and used principal coordinates analysis (PCoA; classical multidimensional scaling, first two axes) to ordinate the data. We tested group-level variation using PERMANOVA (adonis2; 4,999 permutations), with disease status as the main factor and age and sex as covariates. For the paired untreated-versus-treated comparison, we excluded sex, as it does not vary within subjects, resulting in a model with group and age. We assessed significance using exact subject-restricted permutations that swapped the two timepoints within each subject. For the unpaired untreated-versus-control and treated-versus-control comparisons, we used post hoc tests with BH FDR correction. In each ordination, we connected paired samples from the same child with a line to show within-subject changes.

The two data types differed only in the feature transform and distance metric appropriate to each. For the microbiome, counts were CLR-transformed (pseudo-count 1e-6), and Aitchison distances (Euclidean distance on CLR values) were computed; multivariate homogeneity of group dispersions was additionally evaluated with a permutation-based dispersion test (betadisper with permutest). For the metabolome, each platform (NMR and GC-MS) was analyzed separately: features that were entirely missing or constant were removed, any remaining missing values were mean-imputed per feature, and features were auto-scaled (z-scored) before Euclidean distances were computed. Because the NMR and GC-MS inputs are already log_2_-scaled, PCoA on these auto-scaled Euclidean distances is equivalent to principal component analysis on the scaled features.

### Cross-block integration

Genus- and species-level microbiome profiles were each integrated with the combined metabolome (NMR + GC-MS) using multi-block sparse partial least-squares discriminant analysis (DIABLO) in the mixOmics package (v6.28.0) (Rohart et al., 2017). Microbiome features were filtered by prevalence (30%) and CLR-transformed; metabolome features were filtered by prevalence (20%) and log_2_-transformed; both datasets were centered and scaled. Samples common to both blocks were aligned (*n* = 27), and a pooled CeD (untreated + treated CeD) versus control outcome was modeled, as the three-way (untreated/treated/control) design was not supported by the multi-block model (Supplementary Data 1). A full design weight of 0.5 was applied between blocks to balance class discrimination against cross-block correlation. The number of variables kept in each block was tuned using leave-one-out cross-validation over a grid of keepX values, minimizing the centroid-distance balanced error rate (BER), and a single discriminant component was retained for the binary model. Component performance was summarized using the leave-one-out cross-validated BER and the block-wise area under the receiver operating characteristic curve (AUC). Full parameter settings and code are provided in the GitHub repository.

### Late fusion elastic-net modeling

Species-level microbiome and a combined metabolome were used for discrimination between pooled CeD (untreated + treated CeD) and controls. Late fusion elastic-net classification was applied using the glmnet package (v4.1.10) (Friedman et al., 2021). Individual regularized logistic models were trained for each data type (microbiome and metabolome), with hyperparameters (elastic-net mixing parameter α and penalty λ) tuned through inner 3-fold cross-validation over an α grid of (0, 0.25, 0.5, 0.75, 1) and the glmnet λ path, selecting the combination that minimizes inner cross-validated misclassification. Predicative performance was evaluated using stratified 4-fold cross-validation with 5,000 repeats. Within each outer fold, late fusion aggregated block-specific out-of-fold probabilities using weights determined by inner cross-validation performance, and the classification threshold was tuned to maximize balanced accuracy. Model performance was characterized by sensitivity, specificity and balanced accuracy, along with receiver operating characteristic area under the curve (ROC-AUC), precision-recall area under the curve (PR-AUC) and BER, across repeats. The frequency of non-zero coefficients across refits identified stable drivers (features retained in at least 50% of refits). Full parameter settings and code details are provided in the glmnet GitHub script.

## Results

### Cohort demographics

The cohort comprised 30 children divided into three groups based on disease status: untreated CeD (*n* = 11), treated CeD (*n* = 9), and controls (*n* = 10) (Table 1 and; Supplementary Data 2). Treated individuals were paired with samples collected 6 months after commencement of GFD. By 6 months of GFD, all children demonstrated clinical adherence, as confirmed by serological evidence. Anti-tTG IgA levels decreased in every resampled child (median 88 U/mL at baseline to 2 U/mL at 6 months). However, four of nine children remained above the positive threshold ( > 10 U/mL) at 6 months, which is consistent with an expected but incomplete serological response during this period. The mean age is comparable across groups (10.5 ± 2.9 years overall).

**TABLE 1:** Population demographics across groups of children with CeD (untreated + treated CeD) vs. children without CeD (control group).

Group	Count	Age mean ± SD	Sex (male/female)	Anti-tTG IgA Median [range]
Untreated	11	10.7 ± 2.9	7/4 (64%/36%)	88 [7.5 – > 150]
Treated	9	10.7 ± 2.9	6/3 (67%/33%)	2 [ < 1 – 78]
Control	10	10.2 ± 2.8	3/7 (30%/70%)	0.25 [n.d – 3]
Total	30	10.5 ± 2.9	16/14 (53%/47%)	

Untreated CeD individuals represent patients at the time of diagnosis; treated CeD individuals represent those same individuals following 6 months of GFD adherence; and controls represent patients with negative CeD confirmed by both negative serology and duodenal biopsy. Values are n or mean ± standard deviation (*SD*) or fractions and percentages. Anti-tissue transglutaminase IgA (anti-tTG IgA, U/mL) is summarized as the median [range] at the time point each group represents (untreated CeD and control at baseline; treated CeD after 6 months of GFD). Censored results were replaced with fixed values before calculating medians [not detected (n.d) = 0; < 1 = 0.5; < 3 = 1.5; > 150 = 150 U/mL]; the shown ranges retain the original censored notation. Per-participant baseline and 6-month anti-tTG IgA values are available in Supplementary Data 2.

### Gut microbiome structure shows limited global separation, but stage-associated taxonomic changes

**FIGURE 1:**
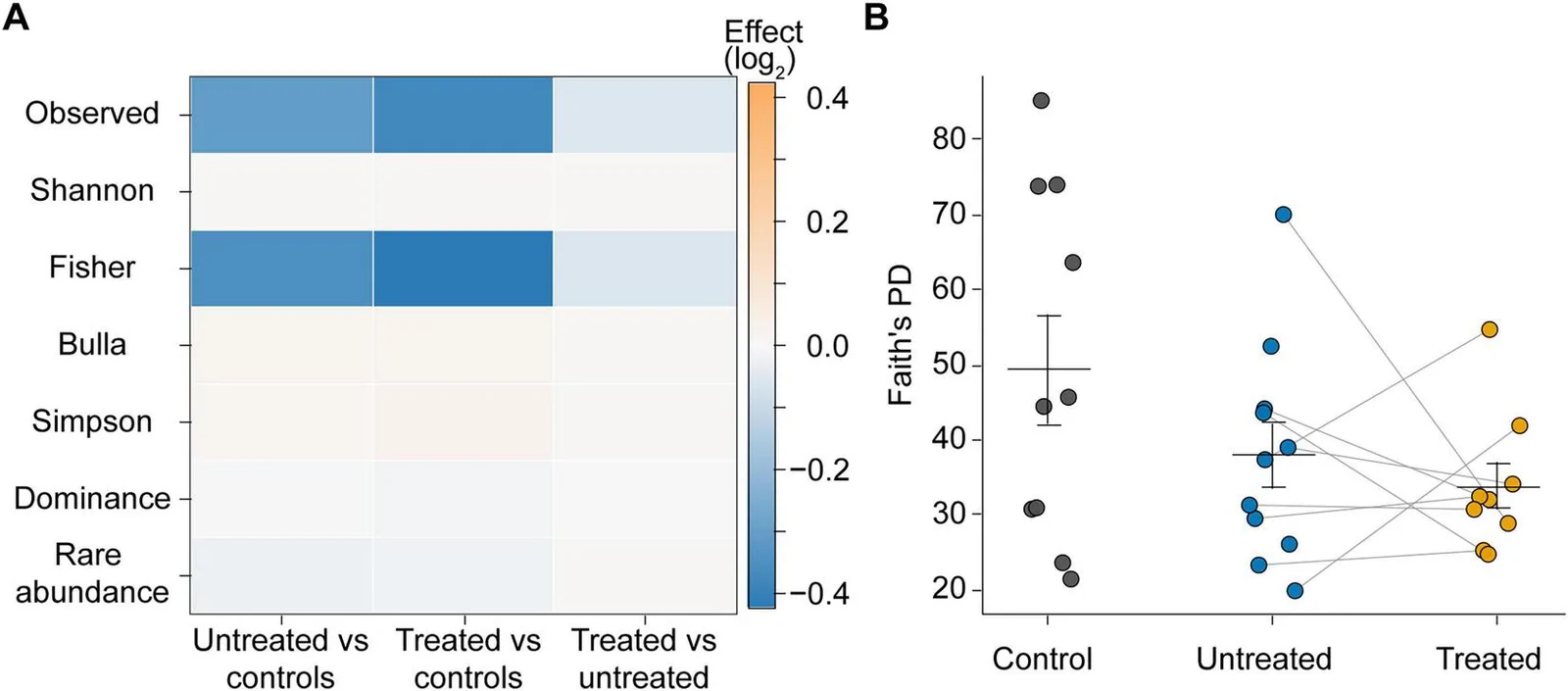
Alpha diversity and phylogenetic diversity in children with untreated CeD, treated CeD, and controls. **(A)** Heatmap showing adjusted log_2_ estimates from the primary linear mixed-effects models for seven alpha diversity metrics across the three comparisons. **(B)** Faith’s PD for individual samples, with points colored by group, gray lines linking paired untreated and treated samples from the same participant, and black summary bars showing mean ± *SE*.

First, the disease-related effects on gut microbiome diversity were defined. Across the alpha diversity and phylogenetic diversity panel, no metric differed significantly between the groups, and the bootstrapping resampling analyses did not identify any metric with a stable non-zero effect (Figure 1 and Supplementary Data 3). Nonetheless, the direction of change was broadly consistent across related richness metrics. Fisher’s diversity and observed richness both trended lower in untreated and treated CeD patients relative to controls (Fisher’s diversity: untreated: estimate = –0.35, standard error (*SE*) = 0.39, *p* = 0.38; treated: estimate = –0.42, *SE* = 0.43, *p* = 0.34; observed richness: untreated: estimate = –0.31, *SE* = 0.35, *p* = 0.39; treated: estimate = –0.38, *SE* = 0.38, *p* = 0.35) (Figure 1A). Similarly, Faith’s PD is lower in both disease groups compared with controls (untreated: estimate = –11.07, *SE* = 9.51, *p* = 0.26; treated: estimate = –13.46, *SE* = 9.48, *p* = 0.18) (Figure 1B). Paired samples show agreement in the direction of effects; however, the transition from untreated to treated CeD appears to reduce the magnitude of change. Taken together, the alpha diversity results suggest a weak, richness-related contraction in CeD, rather than a global diversity deficit.

**FIGURE 2:**
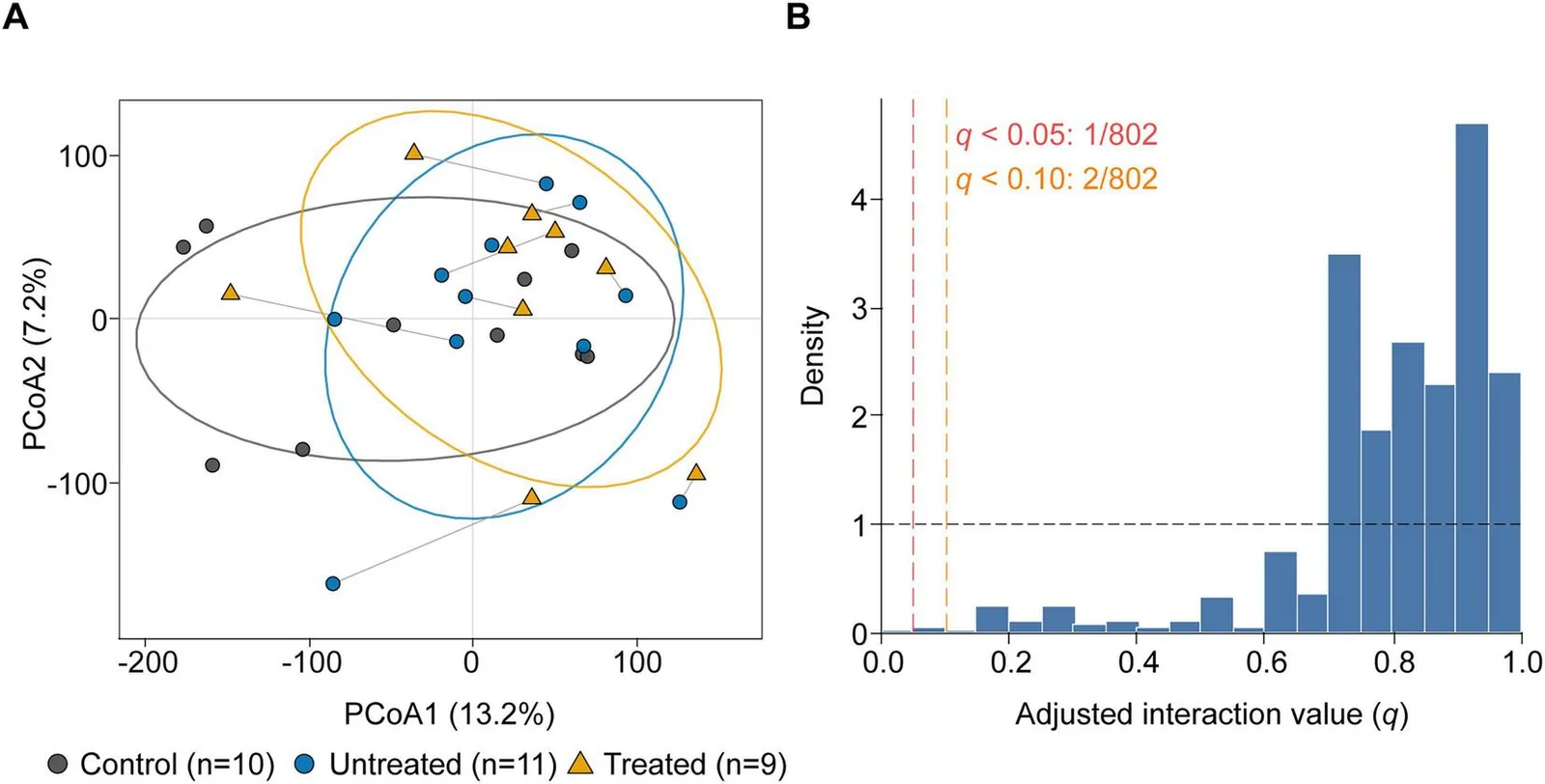
Beta-diversity ordination of gut microbial community composition and interaction sensitivity analysis. **(A)** PCoA of CLR-transformed taxonomic profiles. Points represent individual samples and are colored by group; gray lines connect paired untreated and treated CeD samples from the same participant, and ellipses indicate within-group dispersion. **(B)** Interaction sensitivity bar plot showing BH FDR-adjusted *q*-values across all interaction tests, with thresholds at *q* = 0.05 (red) and *q* = 0.10 (orange).

Next, we defined and analyzed beta diversity across the groups by (PCoA, which does not differentiate by disease group in the multivariable PERMANOVA (Group: *R*^2^ = 0.066, *F* = 0.97, *p* = 0.541) (Figure 2A). Disease status accounted for the largest proportion of variance among the tested covariates, but the limited statistical support suggests high within-group dispersion and limited power. While host covariates accounted for comparable variation with stronger evidence of association, particularly age (Age: *R*^2^ = 0.044, *F* = 1.30, *p* = 0.074), sex explained less variation (Sex: *R*^2^ = 0.038, *F* = 1.11, *p* = 0.227). Post hoc pairwise comparisons explained 3–7% of the variation; none reached statistical significance, although the treated CeD group had the largest effect size (PERMANOVA: *R*^2^ = 0.07, *p* = 0.10). Group dispersions did not differ significantly in any contrast (betadisper, all *p* ≥ 0.28), indicating that the absence of a location effect is not masked by differences in within-group spread (Supplementary Data 3).

Next, pre-specified interaction analyses were conducted to assess whether key metadata influenced the observed effects. This involved testing the Group × Sex and Group × Age interaction terms across alpha diversity, Faith’s PD, beta diversity, metabolomics, and selected 16S rRNA taxon models (Figure 2B and Supplementary Data 3). Of the 802 interaction tests, with BH FDR correction applied within each analysis comparison interaction group, only one interaction reached *q* < 0.05 (Genus: *Erysipelatoclostridium*, Group × Sex, paired treated-versus-untreated, *q* = 0.016), and two reached *q* < 0.10 (Faith’s PD, Group × Age, paired treated-versus-untreated, *q* = 0.069; Aitchison beta diversity, Group × Age, paired treated-versus-untreated, *q* = 0.068). Overall, these findings suggest limited evidence of significant effect modification by Group × Sex or Group × Age across the analyzed outcomes.

**FIGURE 3:**
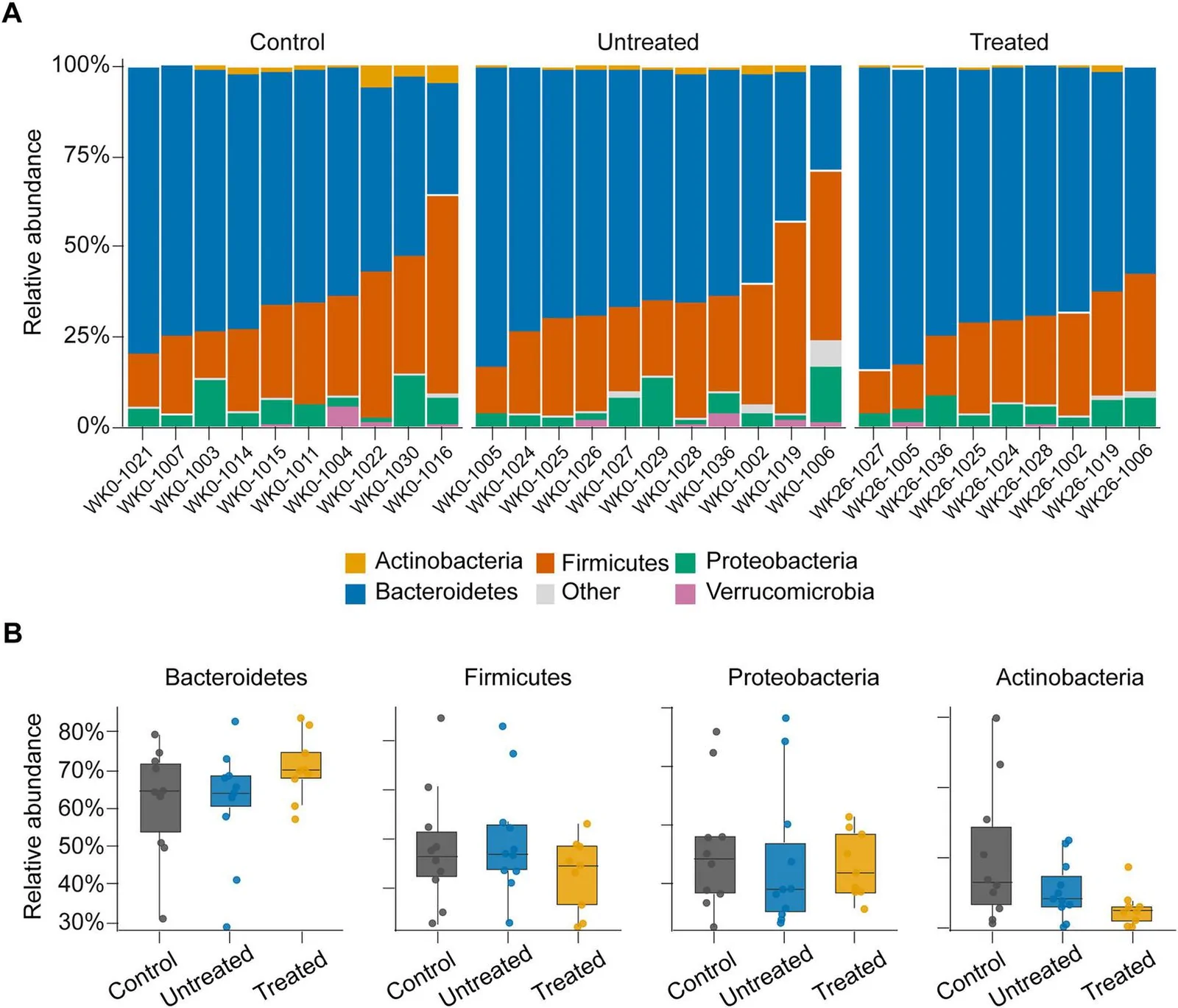
Phylum diversity in children with untreated CeD, treated CeD, and controls. **(A)** Phylum diversity in samples expressed as relative abundance %. **(B)** Boxplots of the relative abundance of the four major phyla colored by group.

Phylum-level community structure was next analyzed and found to be largely conserved, with modest changes across the dominant phyla (Figure 3A and Supplementary Data 3). Unpaired models adjusting for host covariates found no evidence of phylum-level differences between controls and both untreated and treated CeD following BH FDR correction (untreated CeD vs. control, *q* ≥ 0.50 across phyla; treated CeD vs. control, *q* > 0.63). Directionally, untreated CeD trends toward higher Firmicutes (Estimate = 0.066, *q* = 0.51), lower Proteobacteria (Estimate = –0.052, *q* = 0.51) and Bacteroidetes (Estimate = –0.039, *q* = 0.62) (Figure 3B). Treated CeD shows a different pattern, trending toward higher Bacteroidetes (Estimate = 0.050, *q* = 0.74) and reduced Actinobacteria (Estimate = –0.046, *q* = 0.64). Paired comparisons identified a reduction in Firmicutes (Estimate = –0.098, *q* = 0.049) and an increase in Bacteroidetes (Estimate = 0.103, *q* = 0.051), while other dominant phyla show no evidence of change (*q* > 0.40) (Figure 3B). Both paired shifts were supported by the bootstrap sensitivity analysis, being highly directionally stable (bootstrap sign consistency = 1 for both) and with 95% bootstrap intervals excluding zero (Firmicutes: –0.157 to –0.050; Bacteroidetes: 0.036–0.172). Together, this shows that, despite a generally conserved phylum-level structure, early GFD treatment consistently reduces Firmicutes and increases Bacteroidetes.

**FIGURE 4:**
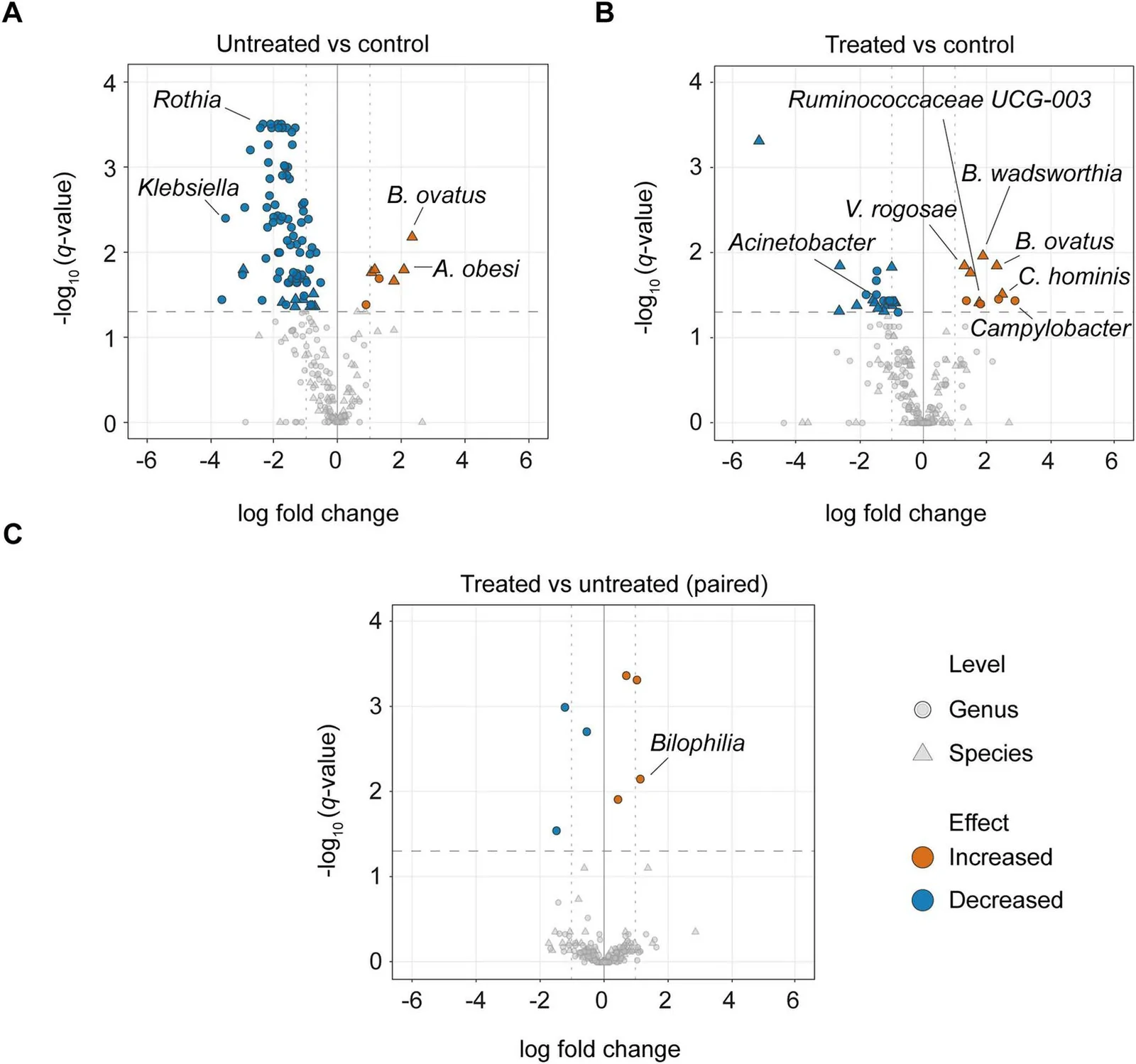
Differentially abundant taxa at both genus and species levels between children with untreated CeD, treated CeD, and controls. Volcano plots show the log fold change against -log_10_ (*q* value) for every tested taxon in three contrasts: **(A)** untreated CeD vs. control, **(B)** treated CeD vs. control, and **(C)** treated vs. untreated CeD (paired). Taxa level is denoted by point shape: genus, circle; species, triangle. Colored points are significant at *q* < 0.05 (orange = increased, blue = decreased in the first-named group); gray points are non-significant. The dashed horizontal line marks *q* = 0.05, and dotted vertical lines mark log fold change = 1. Only taxa that are both statistically significant and robust to resampling are labeled, defined as *q* < 0.05, bootstrap selection frequency ≥ 0.55, a bootstrap confidence interval of the effect estimate excluding zero (stable direction), and a consistently top-ranked effect (30%) across resamples; other significant taxa are shown unlabeled.

Next, we performed differential abundance analysis to identify taxa shifted across groups, revealing a broad taxonomic disruption during untreated CeD, alongside a smaller, more targeted set of changes that persisted or emerged following early GFD treatment (Figures 4A–C and Supplementary Data 4). To differentiate reproducible signals from numerous features that are only nominally significant, each ANCOM-BC2 contrast underwent a 500-resample bootstrap robustness analysis (with subject-level resampling for the paired comparison). The taxa labeled in Figures 4A–C represent the main findings: features that not only achieved BH FDR significance (*q* < 0.05) but also were bootstrap-stable, maintaining a high selection frequency across resamples (≥0.55), a 95% bootstrap LFC interval excluding zero, and a stable bootstrap evidence rank (top 30%). The broader set of taxa that reached *q* < 0.05 but did not meet these bootstrap- and rank-stability criteria is presented as supporting, less strict signals and is not individually labeled.

During untreated CeD, 92 taxa differed from controls at *q* < 0.05, with most representing reductions (Figure 4A). Four of these were bootstrap- and rank-stable, including an increase in species *Bacteroides ovatus* (LFC = 2.34, *q* = 0.007; selection frequency 0.83, bootstrap LFC interval 0.72 to 3.61), and *Alistipes obesi* (LFC = 2.08, *q* = 0.016; selection frequency 0.79, interval 0.01 to 4.21), together with genus-level reductions in *Klebsiella* (LFC = –3.53, *q* = 0.004; selection frequency 0.58, interval –4.22 to –0.003) and *Rothia* (LFC = –2.35, *q* < 0.001; selection frequency 0.71, interval –3.28 to –0.17). The broader set of taxa with *q* < 0.05 indicated widespread depletion and included several well-known gut commensals: *Bacteroides*, *Dialister*, *Subdoligranulum*, *Lactobacillus*, *Escherichia/Shigella*, *Prevotella*, and *Streptococcus*, along with species-level decreases in *Bacteroides stercoris* and *Bacteroides coprocola* (Figure 4A and Supplementary Data 4). These taxa reached significance but did not meet the combined bootstrap- and rank-stability threshold and are therefore interpreted as supporting context rather than primary findings.

Following 6 months of a GFD, the number of differentially abundant taxa decreased to 32 at *q* < 0.05 (Figure 4B). Yet showed the largest number of bootstrap- and rank-stable taxa, with seven constituting the main findings. At the species level, four species showed increases: *Bilophila wadsworthia* (LFC = 1.87, *q* = 0.011; selection frequency 0.76, interval 0.88–3.04), *B. ovatus* (LFC = 2.30, *q* = 0.014; selection frequency 0.72, interval 0.31–3.76), *Veillonella rogosae* (LFC = 1.48, *q* = 0.017; selection frequency 0.63, interval 0.29–4.58), and *Campylobacter hominis* (LFC = 2.48, *q* = 0.031; selection frequency 0.55, interval 0.52–4.70). At the genus level, there were increases in *Campylobacter* (LFC = 2.88, *q* = 0.037; selection frequency 0.56, interval 0.56–5.22) and *Ruminococcaceae UCG-003* (LFC = 1.79, *q* = 0.040; selection frequency 0.59, interval 0.32–3.22), and a decrease in *Acinetobacter* (LFC = –1.48, *q* = 0.021; selection frequency 0.63, interval –2.98 to –0.18). The consistent genus- and species-level signals for *Campylobacter*/*C. hominis*, and the repeated appearance of *B. ovatus* as a stable, disease-related increase in both untreated and treated CeD comparisons, are noteworthy. The broader *q* < 0.05 set identified additional, less-stringent changes that did not pass the combined bootstrap and rank-stability filter, including persistent depletion of *B. stercoris* and *B. coprocola* (both also lowered in untreated CeD), treatment-associated reductions in *Bacteroides plebeius* and *Bifidobacterium bifidum*, and an increase in the genus *Ruminococcaceae UCG-002* (Figure 4B and Supplementary Data 4).

Within-individual paired comparisons of treated versus untreated CeD identified the fewest changes, with only seven reaching *q* < 0.05, all at the genus level (Figure 4C). Of these, only *Bilophila* (LFC = 1.15, *q* = 0.007; selection frequency 0.80, interval 0.83–1.48) was bootstrap- and rank-stable and represented the main finding for this contrast. This increase is consistent with the elevation of *B. wadsworthia* in the treated-versus-control comparison; at the species level, the corresponding paired rise in *B. wadsworthia* did not remain significant after BH FDR correction (LFC = 1.38, *q* = 0.07) (Figure 4C and Supplementary Data 4).

### Metabolomic changes highlight short-chain fatty acids and organic acid shifts across disease states

**FIGURE 5:**
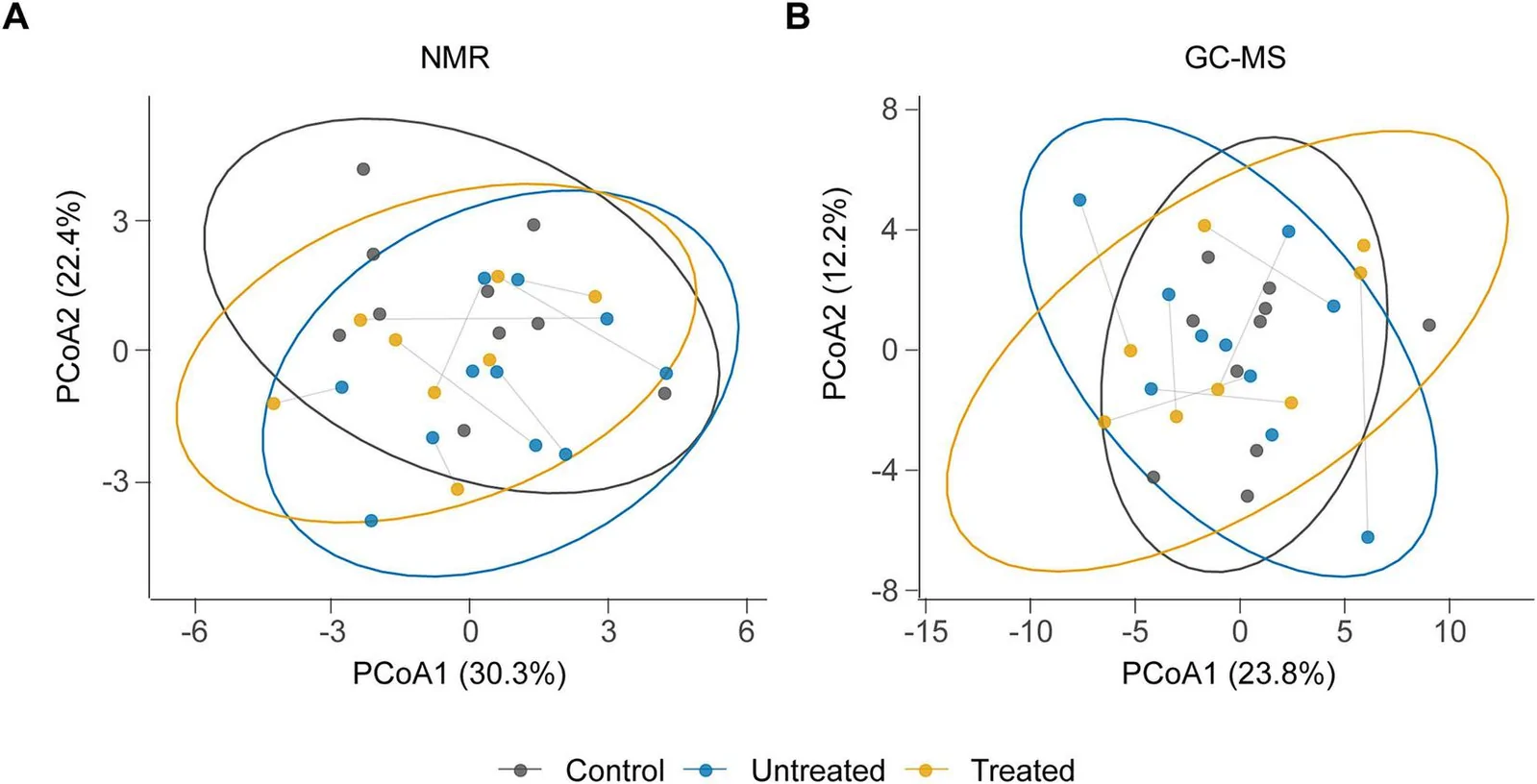
PCoA analysis of NMR and GC-MS metabolites in children with untreated CeD, treated CeD, and controls. **(A)** PCoA ordinations of NMR (15 metabolites) and **(B)** GC-MS (66 metabolites) stool metabolite profiles, calculated using Euclidean distances on auto-scaled (z-scored) feature values. Each point represents a sample, colored by group (control, gray; untreated CeD, blue; treated CeD, orange); gray lines link paired untreated and treated CeD samples from the same participant. Ellipses indicate 95% confidence regions (multivariate *t*-distribution) for each group. The percentage of total variance explained by each axis is shown in parentheses.

Metabolic changes were initially analyzed using PCoA of stool metabolite profiles, computed as Euclidean distances on log_2_-transformed, auto-scaled (z-score) features. Both NMR stool extract profiles (Emwas et al., 2025) and targeted GC-MS profiles showed limited visual separation by group status (Figure 5 and Supplementary Data 5). Consistent with this observation, permutation-based PERMANOVA permutations, adjusted for age and sex, did not identify a significant effect of group status on metabolic variation for either platform (NMR: *R*^2^ = 0.10, *p* = 0.077; GC-MS: *R*^2^ = 0.04, *p* = 0.995). After adjusting for age and sex, individual pairwise contrasts remained non-significant across both platforms. The most notable finding was observed in the paired untreated-versus-treated CeD NMR comparison (*R*^2^ = 0.11, *p* = 0.074). All other contrasts were non-significant (*p* ≥ 0.10 for NMR; *p* ≥ 0.80 for GC-MS). Together, this was consistent with minimal group separation in the overall metabolite community structure identified across both platforms.

**FIGURE 6:**
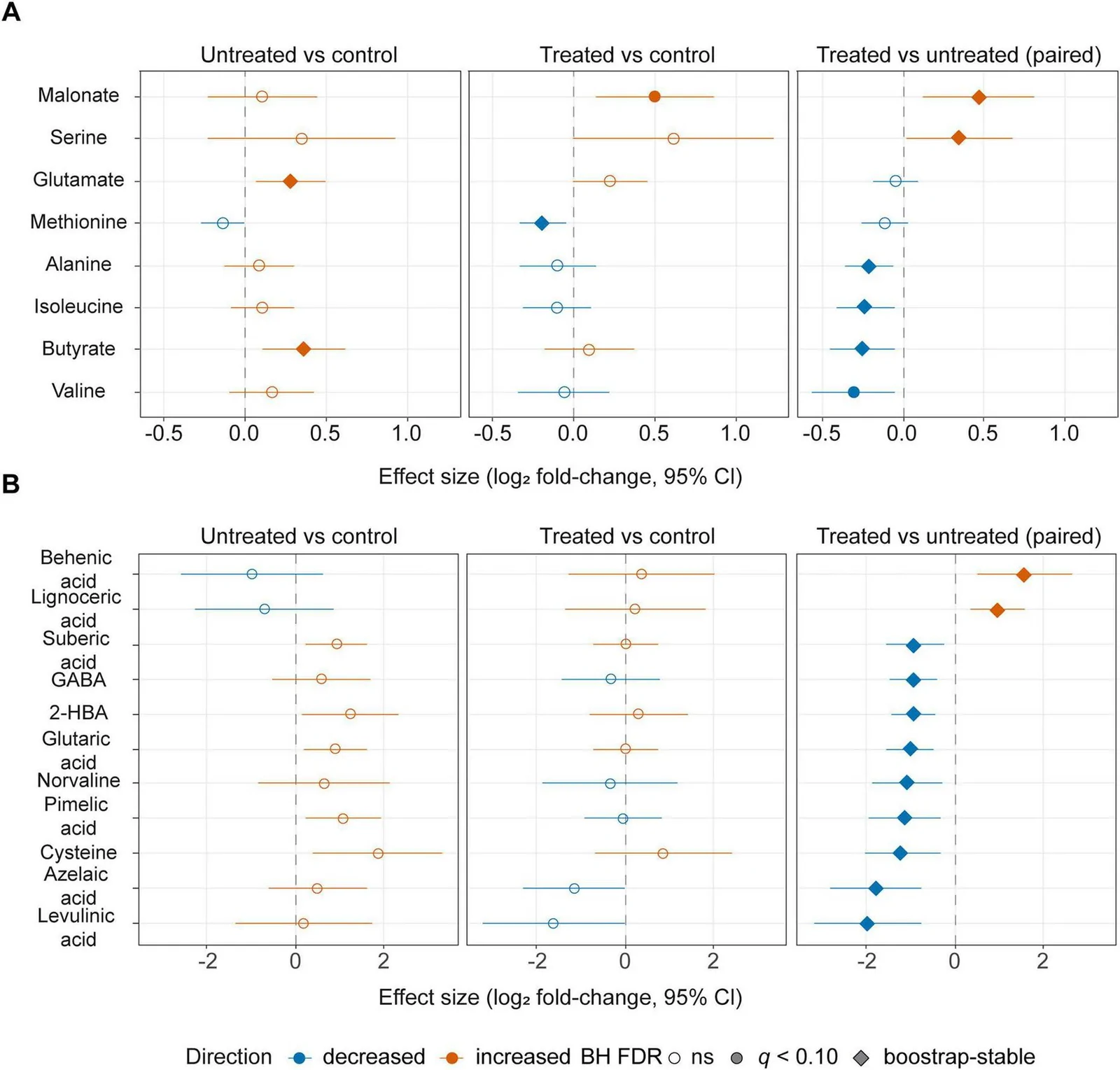
Differential stool metabolites across disease states by NMR and GC-MS in children with untreated CeD, treated CeD, and controls. LMMs’ effect sizes (log_2_ fold-change) for individual metabolites. **(A)** NMR. **(B)** Targeted GC-MS. Each row represents a metabolite; points show the model estimate, and horizontal bars indicate the 95% confidence interval (estimate ± 1.96 × *SE*). Point color indicates the direction of change (blue = decreased, orange = increased), and point shape shows the strength of evidence (open circle = not significant; filled circle = BH FDR *q* < 0.1; diamond = bootstrap-stable, meaning *q* ≤ 0.1 with a 95% cluster-bootstrap interval excluding zero across 500 resamples). The dashed vertical line indicates no change (log_2_ fold-change = 0). Only metabolites with a *q*-value < 0.10 in at least one comparison are displayed. GABA, 4-aminobutyric acid; 2-HBA, 2-hydroxybutyric acid.

LMMs were then used to test for individual metabolite variation across all contrasts, while accounting for host covariates and repeated measures, where applicable. Across all contrasts, both NMR and GC-MS LMMs identified 19 metabolites that differed between groups at a BH FDR *q* < 0.1 and 18 of these remained bootstrap-stable after imposing 500-resample cluster-bootstrap confidence intervals and effect direction consistency; valine (NMR) was the sole exception, leaving a compact set of reproducible, biologically coherent metabolite changes (Figure 6 and Supplementary Data 5).

In the NMR metabolite profiles, untreated CeD and controls differed in two metabolites at BH FDR *q* < 0.1 (Figure 6A and Supplementary Data 5). Both are increased in untreated CeD: butyrate (estimate = 0.36, *q* = 0.08) and glutamate (estimate = 0.28, *q* = 0.08), and both remained bootstrap-stable with 95% cluster-bootstrap intervals entirely above zero. In the paired comparisons (treated vs. untreated CeD), six metabolites differ at BH FDR *q* < 0.1. Four metabolites are lower in treated CeD: valine (estimate = –0.30, *q* = 0.06), isoleucine (estimate = –0.23, *q* = 0.05), butyrate (estimate = –0.25, *q* = 0.05), and alanine (estimate = –0.21, *q* = 0.05), whereas malonate (estimate = 0.47, *q* = 0.05) and serine (estimate = 0.35, *q* = 0.09) increased (Figure 6A and Supplementary Data 5). All five were bootstrap-stable except valine, whose 95% bootstrap interval crossed zero. Treated CeD also differed from controls, with lower methionine (estimate = –0.20, *q* = 0.06), which was bootstrap-stable, and higher malonate (estimate = 0.50, *q* = 0.06), which did not meet the stability criterion (Figure 6A and Supplementary Data 5). Together, these bootstrap-stable changes define a coherent fermentation and amino-acid axis (butyrate, glutamate, branched-chain amino acids, alanine, serine, and malonate) that is elevated in untreated CeD and remodeled after 6 months of GFD (Supplementary Data 5).

Targeted GC-MS profiling was used to provide complementary metabolite coverage and to assess group differences (Figure 6B and Supplementary Data 5). LMMs were then employed to evaluate individual metabolites, while adjusting for host covariates and repeated measures where relevant. Comparisons against controls did not reveal any metabolites that were significantly different after BH FDR correction (*q* < 0.1). Due to the modest cohort size and the consequent limited power for cross-sectional comparisons, the following nominal differences (*p* < 0.05) are presented solely as hypothesis-generating indicators and are not regarded as confirmed findings. Relative to controls, untreated CeD showed nominal increases in several dicarboxylic acids and redox-related metabolites, including adipic acid, glutaric acid, pimelic acid, suberic acid, cysteine, glutamic acid, glutathione, and nicotinic acid, along with nominal decreases in certain saturated fatty acids (e.g., palmitic and arachidic acids) (Supplementary Data 5). In treated disease compared to controls, nominal differences comprised higher levels of adipic acid and malic acid, and lower levels of several saturated fatty acids and organic acids (Supplementary Data 5).

Paired comparisons identified 11 metabolites that were significantly different at a BH FDR *q* < 0.1, and all 11 remained bootstrap-stable, with 95% cluster-bootstrap intervals excluding zero across 500 resamples (Figure 6B and Supplementary Data 5). Treatment was associated with reduced 2-hydroxybutyric acid (2-HBA) (estimate = –0.93, *q* = 0.008), glutaric acid (estimate = –1.00, *q* = 0.008), 4-aminobutyric acid (GABA) (estimate = –0.93, *q* = 0.011), azelaic acid (estimate = –1.79, *q* = 0.011), levulinic acid (estimate = –1.96, *q* = 0.021), cysteine (estimate = –1.18, *q* = 0.051), pimelic acid (estimate = –1.13, *q* = 0.051), suberic acid (estimate = –0.88, *q* = 0.051), and norvaline (estimate = –1.08, *q* = 0.053). Conversely, very-long-chain saturated fatty acids increased following treatment: lignoceric acid (estimate = 0.96, *q* = 0.021) and behenic acid (estimate = 1.59, *q* = 0.031) (Supplementary Data 5). Unlike the cross-sectional disease-versus-control GC-MS comparisons, which did not produce any BH FDR-significant metabolites, this paired GC-MS signal was consistently BH FDR significant and reproducible under resampling and is therefore considered a reliable, treatment-responsive set of organic-acid and fatty-acid changes rather than exploratory differences.

### Cross-block integration reveals coherent and reproducible microbiome-metabolite structure

**FIGURE 7:**
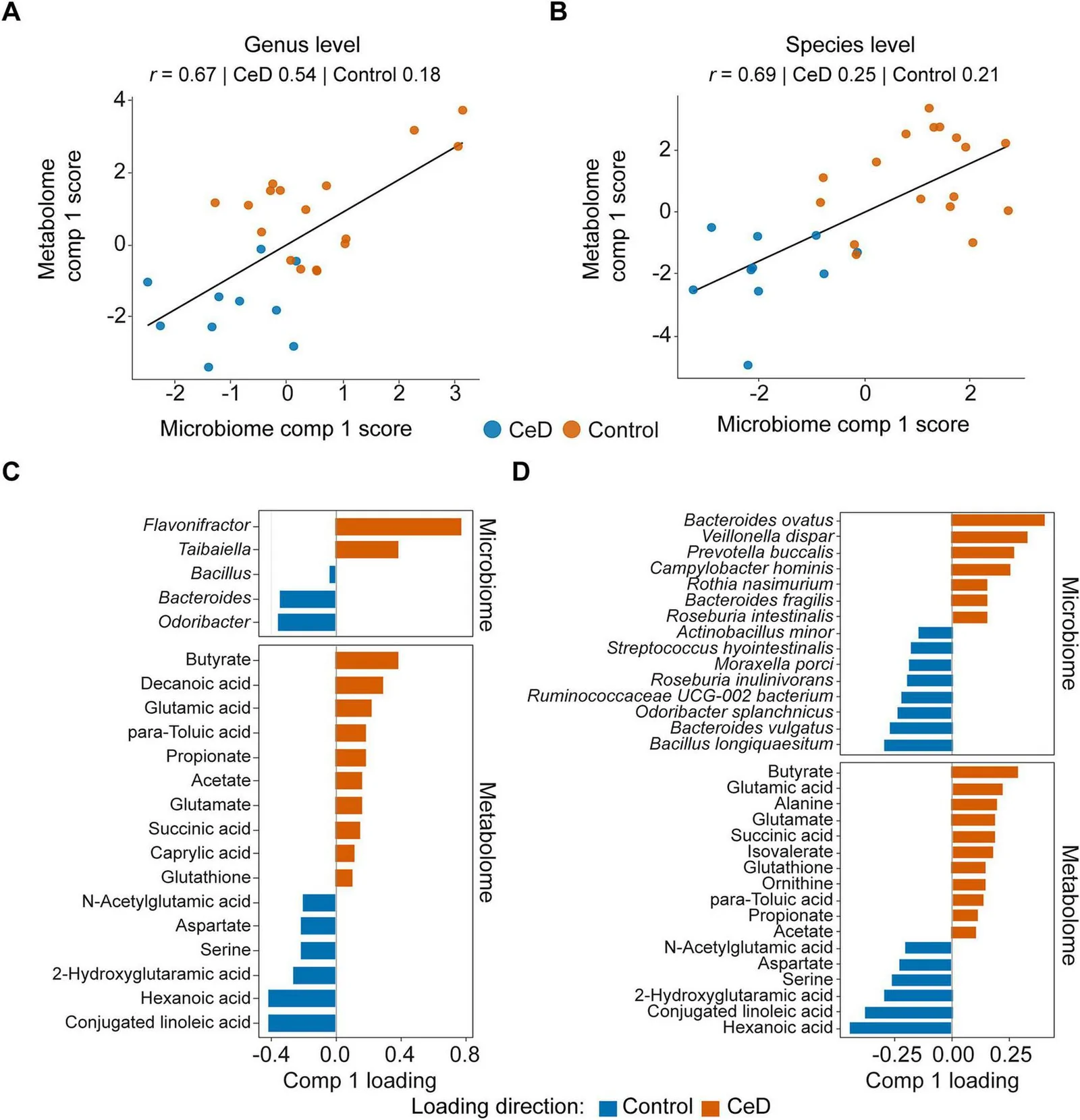
Cross-omic DIABLO axis linking stool microbiome and metabolome in children with CeD (untreated + treated CeD) and controls. Multi-block sparse partial least-squares discriminant analysis was used to find a single latent component that maximally covaries between the microbiome and metabolome blocks while differentiating CeD from controls (*n* = 27; 16S rRNA genus/species CLR abundances and log2 metabolite intensities). **(A,B)** Cross-block sample scores at **(A)** genus and **(B)** species resolution: each point is one child positioned by its microbiome (x) and metabolome (y) component 1 score and colored by disease status, with the black line the linear fit and subtitles reporting the pooled and within-group cross-block correlations. **(C,D)** Component 1 loadings of the leading features in each block are shown at: **(A)** genus level (5 microbial and the 16 top metabolite features selected in total) and **(B)** species level (top 15 microbial and 17 metabolite features selected in total). Bar length is the loading weight, and fill indicates association direction (orange = loads with CeD, blue = loads with controls). Comp, component.

Cross-block integration identified a coordinated microbiome-metabolite structure that distinguishes CeD from controls. The three-way design (untreated CeD, treated CeD, and controls) was not supported by the multi-block model. Neither discriminant component fell below chance (component 1 BER = 0.70, component 2 BER = 0.74; Supplementary Data 1). Because support for three-way discrimination was limited, a pooled CeD (untreated + treated CeD) versus controls model was used. At both the genus and species levels, DIABLO recovered a single discriminant component. The microbiome and metabolome blocks were strongly correlated (genus cross-block *r* = 0.67; species *r* = 0.69). Each child’s microbiome and metabolome component scores co-varied along one shared axis (Figures 7A,B). CeD and control samples separated along this axis, not away from it. The correlation also persisted within groups: CeD samples showed a stronger association at the genus level (CeD *r* = 0.54, control *r* = 0.18) and a weaker association at the species level (CeD *r* = 0.25, control *r* = 0.21). This suggests cross-block coupling is reinforced by, but not solely an artifact of, the disease contrast along the axis.

As a classifier, the shared component was modest: the leave-one-out BER was 0.48 at the genus level and 0.40 at the species level, with the species model performing better. In-sample block discrimination was high (genus AUC = 0.85 microbiome, 0.89 metabolome; species AUC = 0.96 microbiome, 0.91 metabolome; all *p* < 0.01) (Supplementary Data 1). The modest cross-validated BER and the high in-sample AUC indicate that the component captures coordinated cross-omic structures rather than serving as an accurate diagnostic classifier.

The features that define this axis were consistent at both resolutions. At the genus level (Figure 7C and Supplementary Data 1), CeD-loading *Flavonifractor* and *Taibaiella* were associated with the microbiome side, while *Bacteroides* and *Odoribacter* were associated with the control group. On the metabolome side, the most stable CeD-loading features included butyrate, which was the strongest metabolite driver, along with propionate, acetate, glutamate, glutamic acid, glutathione, and succinic acid. These were opposed by control-loading conjugated linoleic acid, hexanoic acid, serine, and aspartate, all of which were stable across cross-validation.

At the species level (Figure 7D and Supplementary Data 1), this axis appeared again with more detail. CeD-loading taxa included *B. ovatus*, *Veillonella dispar*, *Prevotella buccalis*, and *Campylobacter hominis*, while *Bacteroides vulgatus*, *Odoribacter splanchnicus*, and *Bacillus longiquaesitum* were linked to the control group. On the metabolite axis, butyrate remained one of the main stable CeD-loading features, along with glutamate, glutamic acid, alanine, succinic acid, propionate, and acetate. The same control-loading metabolites, hexanoic acid, conjugated linoleic acid, serine, and aspartate, were again opposed. This shows that butyrate and the broader short-chain fatty acid-glutamate axis are associated with CeD at both taxonomic levels.

Overall, these results show that while CeD cannot be separated with high accuracy, the microbiome and metabolome are aligned along a single, consistent axis from genus to species level. This axis highlights a contrast between CeD-associated taxa and SCFAs, and control-associated *Bacteroides*, conjugated linoleic acid, and medium-chain fatty acids. This supports the idea of structured microbe-metabolite coupling instead of a broad shift in the community.

### Cross-validated classification identifies reproducible microbial and metabolite differences according to CeD status

Having established the module-level cross-omics structure and out-of-sample discriminative signal, subsequent supervised feature-level modeling was employed to identify reproducible contributors to disease status. Using the same pooled CeD framework, late-fusion elastic-net classification was applied to CeD vs. controls with species-level microbiome and metabolome models, as genus-level models showed comparatively poor cross-validated performance (Supplementary Data 6).

**FIGURE 8:**
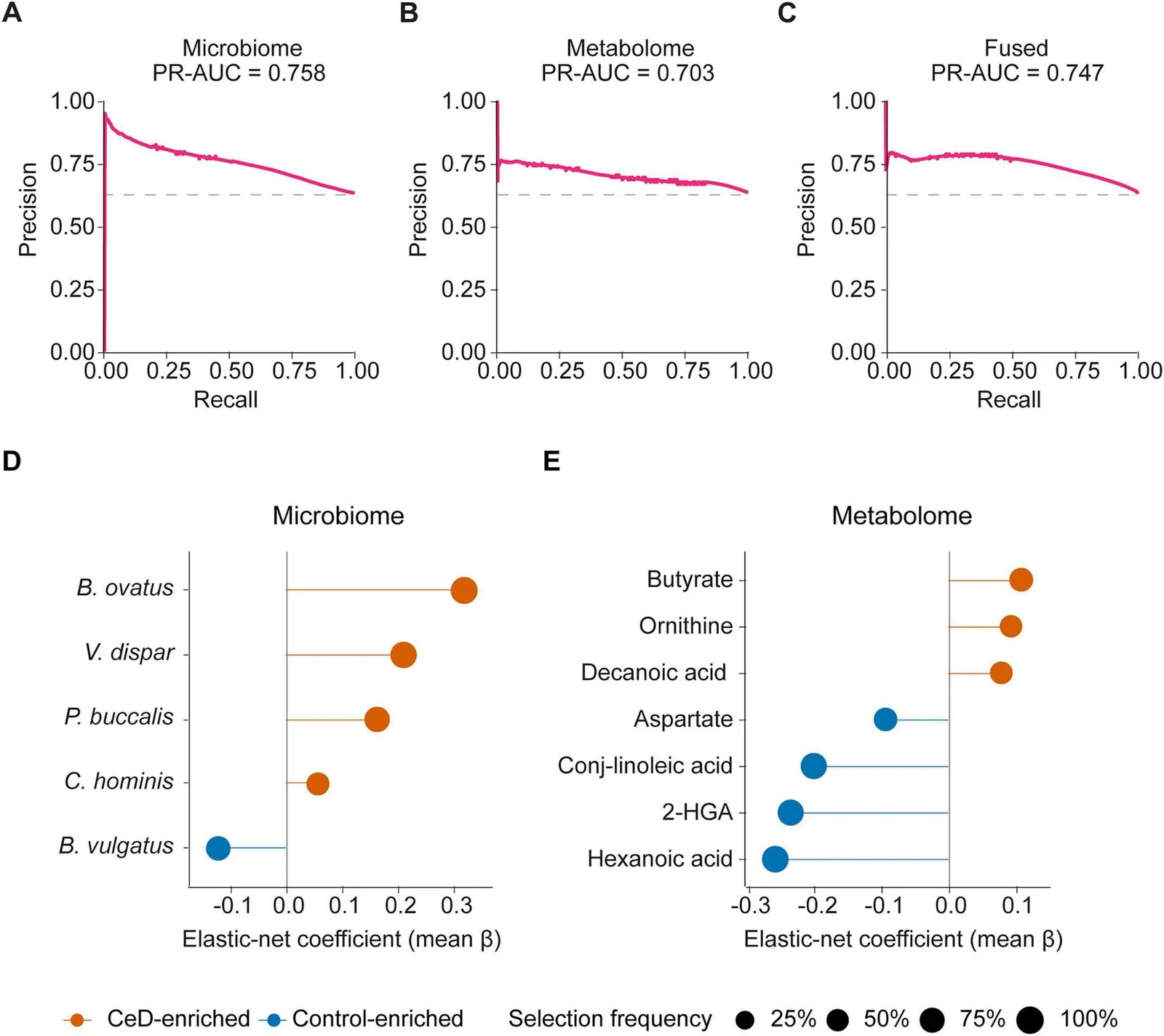
Late-fusion elastic-net classification in children with CeD (untreated + treated CeD) versus controls, and key discriminant features. **(A–C)** Out-of-fold precision–recall (PR) curves for binomial elastic-net models trained on the microbiome (species-level) block **(A)**, metabolome block **(B)**, and the late-fusion model combining block-specific probabilities **(C).** The dashed gray line indicates the baseline for class prevalence. Panel annotations display the pooled PR-AUC calculated from the combined out-of-fold predictions across repeated cross-validation; the curve shown is the corresponding pooled PR curve. **(D,E)** Most consistent features driving discrimination in the microbiome **(D)** and metabolome **(E)**. Point positions show the mean elastic-net coefficient (β) across outer fits; β > 0 indicates CeD-enriched and β < 0 indicates control-enriched features. Point size reflects selection frequency. Conjugated linoleic acid, Conj-linoleic acid; 2-Hydroxyglutaramic acid, 2-HGA.

At species resolution, both single-block models discriminated CeD from controls, with the microbiome block showing the strongest and most consistent discrimination: median BER = 0.424 (IQR = 0.374–0.482), median ROC-AUC = 0.659 (IQR = 0.558–0.729), and median PR-AUC = 0.791 (IQR = 0.738–0.840) (Figure 8A). The metabolome retains a modest signal (median BER = 0.456 (IQR = 0.406–0.506); median ROC-AUC = 0.612 (IQR = 0.541–0.671); PR-AUC = 0.733 (IQR = 0.687–0.779) (Figure 8B). Late fusion performed at least as well as the best single block on every metric and achieved a modest improvement in error rate and ROC-based measures (fused median BER = 0.415, IQR = 0.356–0.465; median ROC-AUC = 0.682, IQR = 0.612–0.741), while its precision–recall performance was comparable to the microbiome block (PR-AUC = 0.776, IQR = 0.730–0.821), indicating that the microbiome and metabolome carry partly complementary information for CeD–control discrimination in this cohort (Figures 8A–C and Table 2).

**TABLE 2:** Cross-validated performance of late-fusion elastic-net models for CeD (untreated + treated CeD) versus controls.

*Block*	*Microbiome*	*Metabolome*	*Fused*
Sensitivity	0.47	0.48	0.49
Specificity	0.68	0.60	0.68
Balanced accuracy	0.58	0.54	0.59
PR-AUC median	0.791	0.733	0.776
PR-AUC IQR	0.738–0.840	0.687–0.779	0.730–0.821
ROC-AUC median	0.659	0.612	0.682
ROC-AUC IQR	0.558–0.729	0.541–0.671	0.612–0.741
BER median	0.424	0.456	0.415
BER IQR	0.374–0.482	0.406–0.505	0.356–0.465

Performance metrics are summarized for microbiome-only (species-level), metabolome-only, and late-fused models combining out-of-fold probabilities. Sensitivity, specificity, and balanced accuracy are computed at the balanced-accuracy–optimized threshold from repeated cross-validation; PR-AUC, ROC-AUC, and BER are reported as median values with interquartile ranges (IQR) across repeats.

Thresholded operating characteristics are consistent with these summary metrics across blocks. Using the balanced-accuracy-tuned threshold, the species-level microbiome achieved sensitivity = 0.47, specificity = 0.68, and balanced accuracy = 0.58, compared with the metabolome, which achieved sensitivity = 0.48, specificity = 0.60, and balanced accuracy = 0.54. Fusion produced sensitivity = 0.49, specificity = 0.68, and balanced accuracy = 0.59 (Table 2). Stability analysis of the elastic-net coefficients identifies a compact set of reproducible drivers (Figures 8D,E): the microbiome signal was dominated by *B. ovatus* (prop selected = 0.94; odds ratio (OR) = 1.37; CeD-up), *V. dispar* (0.89; OR = 1.23; CeD-up), and *P. buccalis* (0.78; OR = 1.17; CeD-up), while the metabolome drivers are primarily control-enriched (e.g., hexanoic acid, conjugated linoleic acid, and 2-hydroxyglutaramic acid, all prop selected ≥ 0.85; OR ∼ 0.77–0.82; Control-up). Overall, the species-level microbiome profile offers the strongest single-block discrimination signal, and late fusion with the metabolome provides a modest additional gain, supporting a modest but complementary discrimination interpretation with stable drivers rather than a high-performance diagnostic claim (Figure 8 and Table 2).

## Discussion

This study characterized the stool microbiome and metabolome of children with CeD in Canterbury, New Zealand, before and after 6 months of GFD treatment. This population exhibits a substantial local disease prevalence (∼ 1 in 82) (Cook et al., 2000; Cook et al., 2004; Kho et al., 2015). Three principal findings emerged from the multi-omics analyses. First, CeD did not display a broad dysbiotic signal but instead demonstrated consistent changes within a stable core of taxa and metabolites, while the broader microbial community and metabolome remained largely unchanged. Second, these stable changes involved both *Bacteroides* diversity and their roles in multi-omics analyses for disease discrimination. Notably, *Bacteroides ovatus* was associated with both active and treated CeD, alongside distinct metabolite changes, including elevated butyrate and altered redox-related metabolites, most of which returned to control levels following a GFD. Third, single-omic separation was weak, and classification accuracy was modest (PR-AUC = 0.731–0.791, ROC-AUC = 0.66–0.68). However, the microbiome and metabolome were aligned along a single, reproducible cross-omic axis, indicating a structured relationship rather than a broad collapse in this cohort.

The observed stability of the gut microbiome community represents a significant finding. Measures such as alpha diversity, phylogenetic diversity, and overall composition revealed minimal differences between groups. This observation is consistent with previous challenges in identifying clear taxonomic markers for CeD (Leonard et al., 2020; Leonard et al., 2021; Prendergast et al., 2026; Zafeiropoulou et al., 2020) and supports the notion that CeD lacks the broad dysbiotic pattern observed in other immune-related diseases (Turjeman et al., 2023). The combination of bootstrap resampling with standard significance tests enabled the exclusion of unstable features, thereby retaining a reliable signal. The results indicated that the overall community structure was largely similar between untreated CeD and controls, but shifted following 6 months on a GFD. This finding aligns with a recent pediatric study, which reported that the gut microbiome resembled controls at diagnosis but changed after a year on a gluten-free diet (Sample et al., 2021). Collectively, these results indicate that both dietary intervention and disease status play critical roles in shaping the gut microbiome in CeD (Bonder et al., 2016; Costigan et al., 2025; Zafeiropoulou et al., 2020).

Within this context, the most reproducible taxonomic signal was a restructuring within *Bacteroides* diversity, rather than a uniform change across the genus. At the genus level, *Bacteroides* exhibited a modest, non-robust reduction in untreated CeD compared to controls and remained unchanged in treated CeD relative to both controls and within-individual paired contrasts. This variability aligns with previous studies reporting contrasting directions in *Bacteroides* abundance at the genus level (Collado et al., 2007; Collado et al., 2009; De Palma et al., 2010; El Mouzan et al., 2022; Milletich et al., 2022). In contrast, reproducible signals were observed at the species level, with individual *Bacteroides* species shifting in opposite directions across groups. This pattern is consistent with a diverse genus comprising specialized foragers of dietary and host glycans, with species occupying distinct nutritional niches (La Rosa et al., 2022; Wexler, 2007). Given that specific *Bacteroides* species have demonstrated both protective effects, such as the production of beneficial compounds like SCFAs, and exacerbating effects on pro-inflammatory states through epithelial stress and gluten proteolysis, the dominant species are directly relevant to CeD pathogenesis (Briliûtë et al., 2019; Caminero et al., 2019; Galipeau and Verdu, 2022; Wexler, 2007).

The most prominent feature was *B. ovatus*, which increased in both untreated and treated CeD relative to controls and was a leading driver of the cross-omic axis and elastic-net classifier. This finding is notable, as *B. ovatus* is generally regarded as beneficial and a next-generation probiotic candidate (Shen et al., 2026). *B. ovatus* has been shown to promote the production of reparative IL-22, reduce colitis in mouse studies (Ihekweazu et al., 2021), and serve as an engineering model for live therapeutics in inflammatory bowel disease (Hamady et al., 2010). Previous studies have found *B. ovatus* mostly unchanged or reduced in response to untreated CeD (El Mouzan et al., 2022; Sánchez et al., 2012), although increases have been seen in response to GFD treatment (Costigan et al., 2025; Slager et al., 2025). One interpretation is ecological rather than pathogenic: *B. ovatus* is a metabolic generalist capable of growth across a wide range of pH, bile, and oxidative conditions (Fultz et al., 2021), which may allow expansion in a perturbed gut. However, its increase in CeD does not necessarily indicate a benign role, as previous literature shows *B. ovatus* can elicit systemic IgG and IgA responses in inflammatory bowel disease (Saitoh et al., 2002). Such contrasting responses may be explained by strain-level differences, as *Bacteroides* strains within the same species can exhibit different physiological and pathological phenotypes (Lin et al., 2019). Given that 16S rRNA data cannot resolve strain-level variation, interpretation should focus on the reproducible direction of change, which can be informative prior to mechanistic elucidation. Nonetheless, mechanistic validation remains a priority rather than assuming a straightforward pathogenic role. A similar pattern was observed for *B. fragilis*, a clinically established pathogenic *Bacteroides* (Wexler, 2007), which was enriched in CeD in cross-omic analyses and contains strains with metalloproteases capable of hydrolysing gliadin into immunogenic peptides, increasing epithelial permeability and tumor necrosis factor-α release (Sánchez et al., 2012). In contrast, *B. vulgatus*, which exhibits a protective phenotype against gluten-exposed epithelium through epigenetic reprogramming (Tran et al., 2024) and reduces colitis in mouse models (Liu et al., 2022), was consistently associated with controls. Additional control-associated species included *B. stercoris*, linked to improved metabolic health in high-fat-induced obesity in mice (Ryu et al., 2023), and *B. coprocola*, which modulates neuroinflammation through the production of SCFAs butyrate and acetate (Liu et al., 2026; Wexler, 2007), both of which were decreased in untreated and treated CeD. Collectively, CeD in this cohort showed a shift away from protective *Bacteroides* species toward those with established pathogenic or uncertain roles with this shift retained across early GFD treatment.

The metabolic results resolved into two biologically coherent axes. The first axis reflected a redox and energy-stress signature. Within-individual treatment robustly reduced 2-HBA, cysteine, and a series of dicarboxylic acids (glutaric, azelaic, pimelic, suberic), several of which were nomically increased in untreated CeD, while glutathione and glutamate were also elevated in untreated CeD. These changes are mechanistically grounded: glutathione (a glutamate-cysteine-glycine tripeptide) is the primary antioxidant in the gut, and 2-HBA is a byproduct of glutathione synthesis and a recognized marker of oxidative stress (Circu and Aw, 2012; Gall et al., 2010). The dicarboxylic acids axis can be explained by γ-oxidation, which occurs when the cell’s primary fat-burning pathway (β-oxidation) is overloaded (Ranea-Robles and Houten, 2023). This glutathione-centered redox defense state aligns with documented mucosal oxidative stress in CeD enteropathy (Ferretti et al., 2012; Stojiljković et al., 2012). Because these changes are observed only in within-individual paired comparisons, and not in treated-control comparisons, these reductions likely reflect progressive normalization of an untreated CeD oxidative-stress signature rather than GFD-induced reductions. Importantly normalization in this context means a shift towards control levels, not statistical equivalence to a healthy reference.

The second axis was centered on fermentation. Butyrate was elevated in untreated CeD, anchoring the CeD-associated end of the cross-omic axis alongside other SCFAs (acetate, propionate) and glutamate, all of which have established roles in immunological regulation (den Besten et al., 2013; Furusawa et al., 2013; Roediger, 1982; Smith et al., 2013; Yang et al., 2020). Butyrate serves as the primary fuel source for colonocytes, drives regulatory T cell differentiation, and promotes the production of barrier-protective cytokines (IL-10, IL-22) (den Besten et al., 2013; Furusawa et al., 2013; Roediger, 1982; Smith et al., 2013; Yang et al., 2020). Elevated stool butyrate in untreated CeD should not be interpreted as evidence of excessive fermentation. The directionality of butyrate differences in stool can reflect multiple processes, including altered microbial production, utilization by colonocytes, absorption, or changes in substrate availability due to disease and diet (den Besten et al., 2013; Roediger, 1982). The most plausible explanation is that a damaged mucosa in untreated CeD absorbs less butyrate, leading to higher luminal butyrate levels (Nimri et al., 2022; Pascual et al., 2014). This interpretation is supported by the observed decrease in butyrate levels in the treated CeD within-individual paired comparisons, which did not fall below control levels, consistent with a progressive normalization following early GFD treatment. Previous stool-based studies have reported comparable results (Baldi et al., 2021; Nistal et al., 2012; Tjellström et al., 2005; Tjellström et al., 2013), while a recent study focusing on the duodenal niche found a reduction in butyrate (Wulczynski et al., 2025), highlighting that stool and small-intestinal compartments may differ. The concurrent identification of butyrate and SCFAs as key features across analyses underscores their potential as targets for intervention and disease monitoring.

Not all treatment-associated changes were attributable to normalization. A small subset reflected genuine GFD effects. At the metabolite level, methionine was the only stable finding lower in treated CeD compared to controls, while malonate was higher but not stable in this contrast. The clearest early GFD treatment response was microbial: the bile-tolerant, sulfidogenic pathobiont *Bilophila wadsworthia* increased in treated CeD relative to controls (Sayavedra et al., 2025). *B. wadsworthia* growth is often associated with increased dietary fat via taurine-conjugated bile acids and can aggravate gut-barrier dysfunction and promote systemic inflammation (Feng et al., 2017; Natividad et al., 2018). This is consistent with the higher-fat, lower-fiber profile of the GFD (Gessaroli et al., 2023; Vici et al., 2016). Notably, the genus *Bilophila* was the only taxon both significant and resample-stable in within-individual paired comparisons, a contrast that most directly isolates the effect of treatment, as each child serves as their own baseline. The finding that the most robust within-individual consequence of gluten withdrawal is an increase in a fat-driven, potentially pro-inflammatory pathobiont suggests that the GFD does not simply normalize the gut ecosystem but imposes its own, potentially unfavorable, signature.

It is important to note that this normalization was specific to certain features. We observed clear microbial changes that lasted after 6 months of a GFD, even though some untreated CeD associated metabolites moved closer to control values. This means the treatment response was not the same throughout the gut ecosystem. Overall, these changes show a gradual, transitional pattern. Some features become more like those in healthy controls, others stay mostly the same, and a few are introduced or increased by the diet itself, such as the increase in *Bilophila* linked to higher fat intake (Feng et al., 2017; Natividad et al., 2018). This uneven and partial recovery matches other evidence showing that normalizing the celiac microbiome and its fermentation products takes time, often more than a year on a GFD (Bonder et al., 2016; Costigan et al., 2025; Sample et al., 2021; Zafeiropoulou et al., 2020), mirroring the incomplete mucosal healing that commonly persists beyond the first year of treatment (Catassi et al., 2022; Lindfors et al., 2019).

Overall, these results suggest a clear direction for monitoring that could be valuable in both research and clinical practice, pending further validation. This study highlights a set of stable candidate features for the field: *B. ovatus*, the linked butyrate/SCFA and glutathione-redox patterns, and the GFD-specific *Bilophila* increase. These features should be tested casually in gnotobiotic or *ex vivo* models and subsequently be replicated in larger longitudinal cohorts. Methodologically, the stability filtering and cross-omic integration approach presented here offers a transferable template for extracting reproducible results from modestly sized multi-omics cohorts. Longitudinal monitoring may be the most valuable outcome of this work. Because pediatric CeD diagnosis often involves collecting stool and duodenal biopsies, and monitoring both GFD adherence and treatment efficacy is challenging (Elli et al., 2024; Wieser et al., 2021), the addition of 6- and 12-month resampling, if the features we found to change are validated, could provide vital tools that may transform routine follow-ups into a functional readout of treatment response. This would enable tracking of butyrate/SCFA and redox feature normalization, as well as detection of other inadequacies in GFD adherence, such as *Bilophila* expansion or lack of metabolic recovery not captured by serology. Such detailed monitoring could facilitate more targeted patient-level interventions to address both uncommon disease-associated responses and nutritional deficiencies associated with GFD. These approaches are particularly relevant to Canterbury, where the high disease burden and substantial pool of screen-detected or non-classical CeD create a clear need for functional follow-up tools beyond serology (Cook et al., 2000; Cook et al., 2004; Kho et al., 2015). Incorporating longitudinal stool microbiome–metabolome monitoring into routine pediatric follow-up would enable prospective validation of the stable features identified and help determine whether microbiome-targeted adjuncts could address residual dysbiosis at 6 months.

Limitations of this study include a modest cohort size (*n* = 30), limiting power for cross-sectional contrasts. This can be seen in several GC-MS disease-versus-control differences, which were only nominal, and in the normalization interpretations, which rest partly on a near-null treated-versus-control comparisons. Controls were non-CeD children undergoing endoscopic investigation and, although negative for CeD, may not represent metabolically or microbiologically healthy peers; as such, this may attenuate disease contrasts. The 6-month window sampled here captures early GFD treatment responses rather than complete recovery which can take years instead of months (Catassi et al., 2022; Lindfors et al., 2019). Stool biology also reflects an integrated output of diet, host physiology, and the microbiome instead of a direct readout of mucosal properties (Constante et al., 2022; Galipeau and Verdu, 2022; Wulczynski et al., 2025). We did not assess hepatic function or bile-acid pools in this study. While duodenal biopsy and serology ruled out clear cases of CeD and enteropathy, these methods cannot detect the mild, gluten-responsive liver involvement seen in pediatric CeD. As a result, we cannot rule out this possibility, and it may help explain the bile-acid-linked signals we observed. Placing this study’s results to pediatric CeD cohorts globally, it becomes clear that geography, diet, genetics, and age all have a strong impact on the microbiome signals reported (Leonard et al., 2020; Leonard et al., 2021; Prendergast et al., 2026). Since these factors vary widely across groups, taxonomic signatures are rarely identical across populations, and even similar findings are influenced by local diets and genetic backgrounds. This means that microbiome patterns seen in children are unlikely to apply directly to adults. Finally, the study’s associative design cannot establish causation.

In summary, this multi-omics study involving children from Canterbury, New Zealand, finds that CeD-associated stool biology is best captured as a reproducible set of specific microbe-metabolite changes rather than a single global community shift. The repeated emergence of *Bacteroides* restructuring, differences in butyrate/SCFAs, and glutathione-redox-associated features across analyses, together with stable cross-omics networks, provides a biologically coherent set of hypotheses for future validation. Although this study is from a single New Zealand population inhibiting global generalization its interaction-centric signatures provide mechanistic interactions that can be tested across global pediatric CeD cohorts. Together, these data establish a platform for pediatric CeD multi-omics in Canterbury, where a high disease burden and diverse clinical presentations make this population especially suited to validating the reproducible taxa and metabolites prioritized here.

## Data Availability

The original contributions presented in the study are publicly available. The 16S rRNA gene amplicon sequencing data have been deposited in the NCBI Sequence Read Archive (SRA) under BioProject accession PRJNA1484843 (https://www.ncbi.nlm.nih.gov/bioproject/PRJNA1484843). All other datasets generated and analyzed in this study are included in the article/Supplementary material. All code used in this study is hosted on the GitHub repo (https://github.com/pjp72-Un/CanterburyMultiomicsRepo). Further inquiries can be directed to the corresponding author.
