# Supplementary material for: Integrated multi-omics reveals stable microbe–metabolite relationships in pediatric celiac disease
Source: Front Microbiol. 2026 Jul 27;17:1866060. doi: 10.3389/fmicb.2026.1866060 (PMC13454391; doi:10.3389/fmicb.2026.1866060)
Supplement: Supplementary file 1 [file Supplementary_file_1.pdf]

Supplementary Material

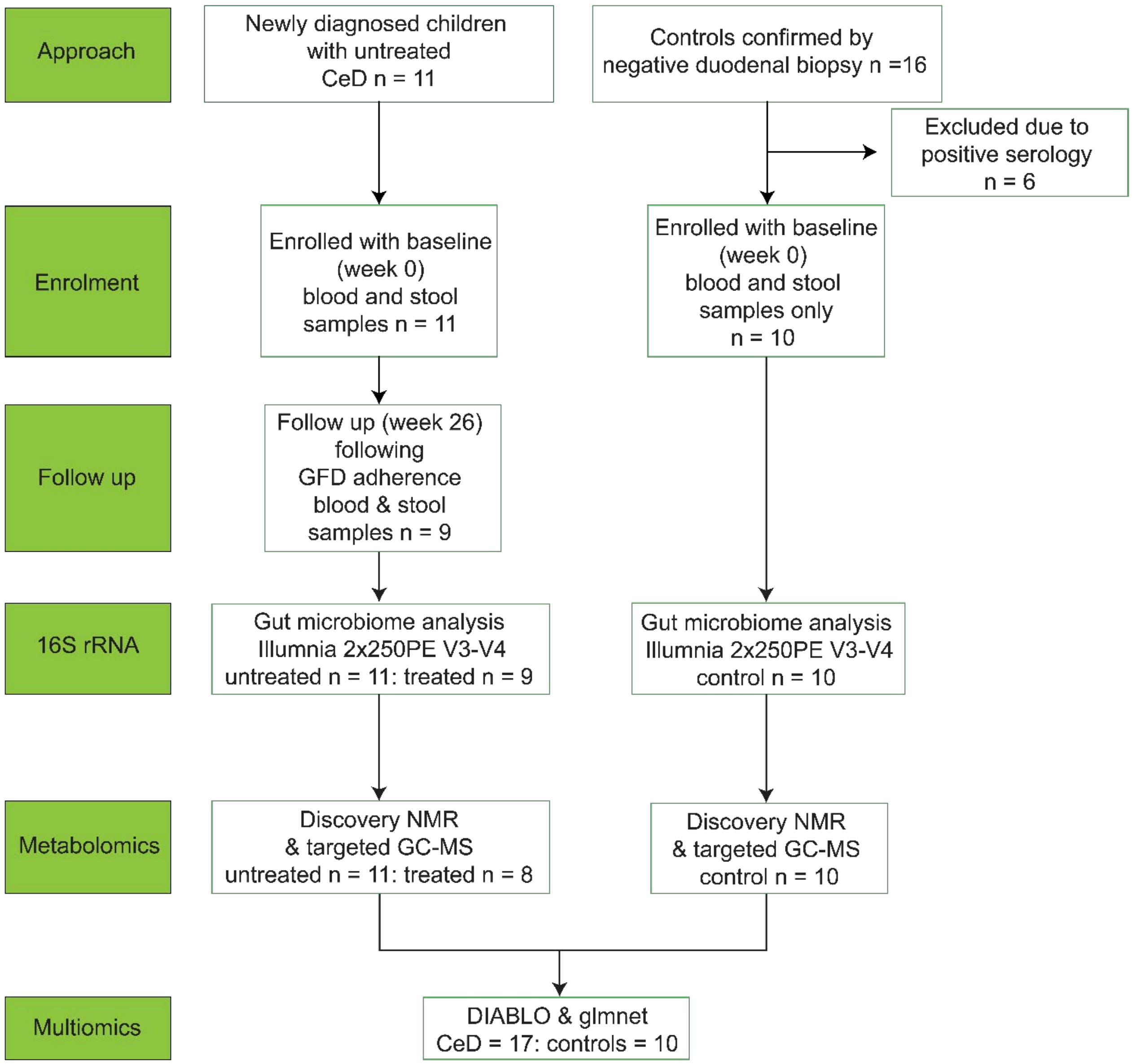

Supplementary Figure 1. Study design and pipeline. GFD, gluten-free diet; 16S rRNA, 16S ribosomal RNA; NMR, nuclear magnetic resonance; GC-MS, gas chromatography-mass spectrometry; DIABLO, multi-block sparse partial least-squares discriminant analysis; CeD, coeliac disease.

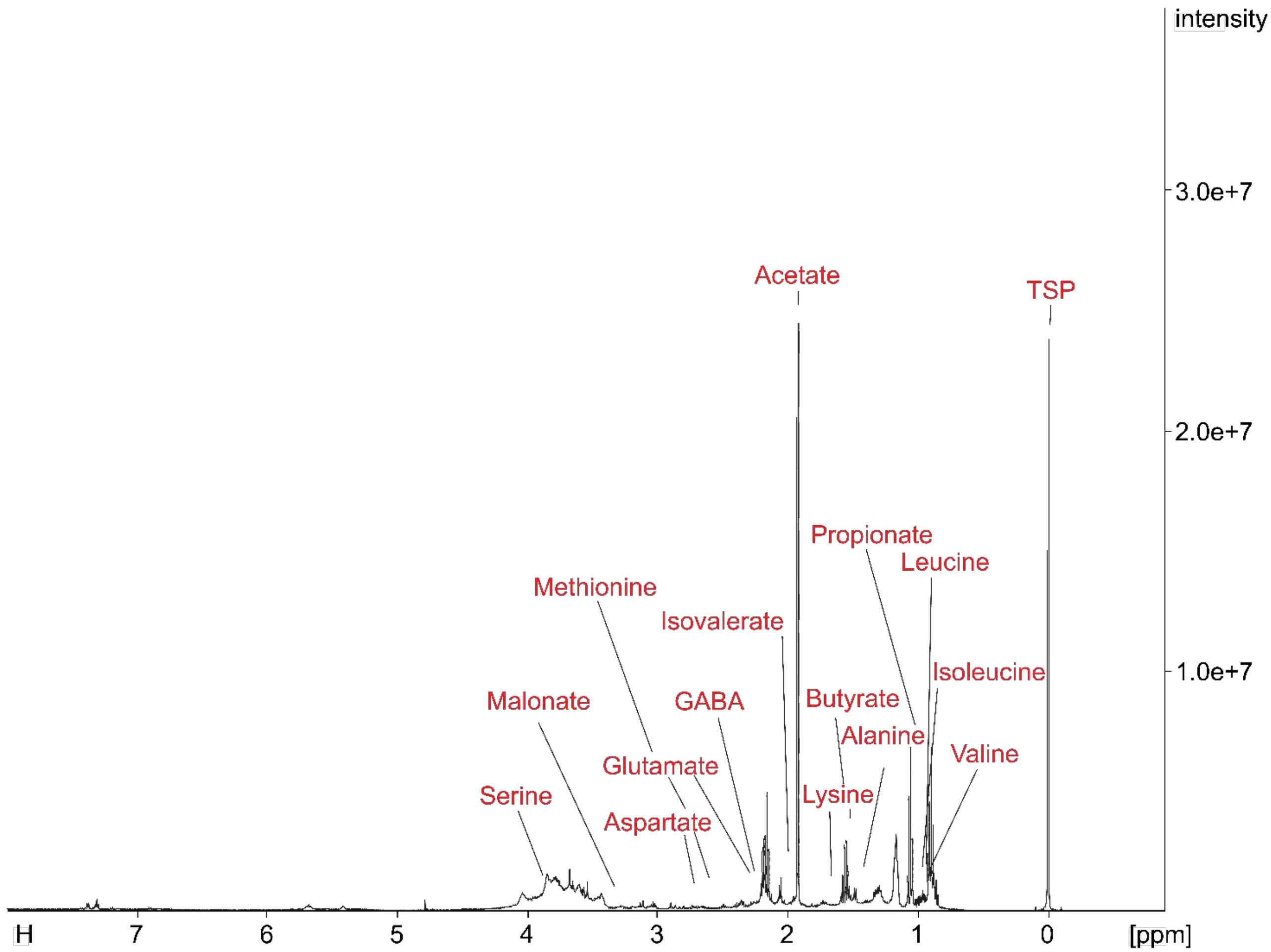

Supplementary Figure 2. One-dimensional NMR spectrum from stool extracts with annotated representative non-overlapping peaks. GABA, 4-aminobutyric acid; ppm, parts per million; TSP, Sodium 3-trimethylsilyl propionate.
